# Determining the Ion Mobility in Perovskite Solar Cells from Impedance Spectroscopy

**DOI:** 10.1021/acsenergylett.5c01690

**Published:** 2025-08-26

**Authors:** Fransien D. Elhorst, Javier E. Sebastián Alonso, Henk J. Bolink, L. Jan Anton Koster

**Affiliations:** † Zernike Institute for Advanced Materials, University of Groningen, Nijenborgh 3, 9747 AG Groningen, The Netherlands; ‡ Instituto de Ciencia Molecular, 16781 Universidad de Valencia, C/Catedrático J. Beltrán 2, 46980 Paterna, Spain

## Abstract

Mobile ions in metal halide perovskites are found to degrade perovskite solar cells (PSCs). Therefore, characterizing their density and mobility is crucial for improving the long-term performance of PSCs. We introduce a formula to determine the mobility directly from impedance spectroscopy. The validity of the formula is confirmed through extensive drift-diffusion simulations varying 38 parameters including mobilities, ion densities, layer thicknesses, and trap densities. All in all, these simulations describe a wide variety of PSCs. Alternative formulas from the literature are also tested but are found to be suboptimal. After validation, we experimentally determine the ion mobility of a methylammonium lead iodide PSC to be 4 × 10^–10^ m^2^ V^–1^ s^–1^. This new formula, which depends on the low-frequency feature of the impedance spectrum, facilitates the precise and straightforward determination of the ion mobility in PSCs.

## Graphical abstract

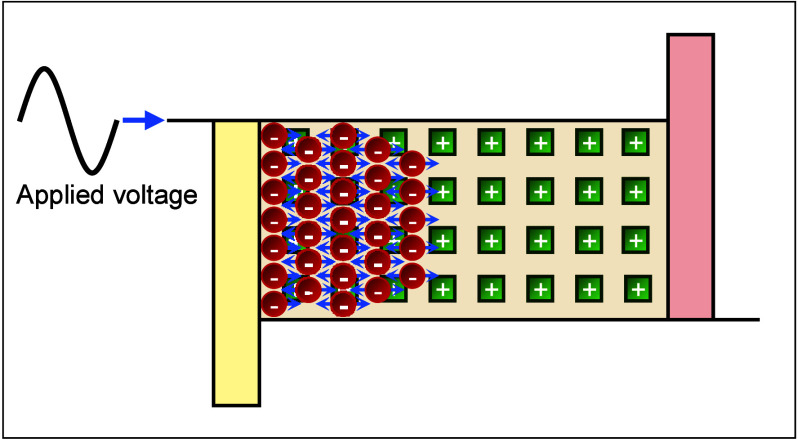

Only in the last 15 years has the power conversion efficiency (PCE) of perovskite solar cells (PSCs) increased from an initial 3.8%^1^ to 26.1% in 2024,^2^ making perovskites the most promising material group to out-compete silicon solar cells. Despite this unprecedented advancement, PSCs still have a major challenge to overcome: their stability. The stability issues of PSCs have been linked to ion motion.^3,4^ Recently, Thiesbrummel et al. even identifies increased mobile ion density as the *dominant* factor.^5^ Since mobile ions screen the internal electric field of the perovskite, they hinder charge carrier extraction, so higher mobile ion densities with aging will result in progressive PCE loss. Therefore, characterizing the ion density and mobility is crucial to improve the long-term PCE. Insight in these physical quantities allows to optimize the perovskite composition,^6^ transport layer properties,^7^ pixel spacing,^8^ etc.

The nature of the mobile species in PSCs is generally thought to be iodide or its vacancies;^9–14^ however, the presence of other dominant and/or additional mobile species are also suggested, such as methylammonium (MA^+^),^15,16^ or H^+^.^17,18^ If different ions diffuse on different time scales, their diffusion coefficients may uncover the mobile species and may explain the broad range of reported ion diffusion coefficients for PSCs in the literature (10^–10^ to 10^–20^ m^2^s^–1^).^5,19–28^ (In this Letter, we use both “ion diffusion coefficient” and “ion mobility” terms and convert between them using the Einstein relation for diffusion of charged particles: *qD*_ion_ = μ_ion_ *k*_B_ *T*, where *q* denotes the elementary charge, *D*_ion_ is the ion diffusion coefficient, μ_ion_ is the ion mobility, *k*_B_ is the Boltzmann constant, and *T* is the temperature.) Thus, there is a clear need for a standardized method that can determine the ion diffusion coefficient both accurately and efficiently.

We put impedance spectroscopy (IS) forward as a promising solution. For most PSCs, the impedance spectra exhibit one low-frequency (LF) feature and one high-frequency (HF) feature.^26,29^ The LF feature is attributed to ion motion,^30^ and the HF feature is attributed to electronic processes such as bimolecular and trap-assisted recombination.^29^

Traditionally, impedance measurements are analyzed using equivalent circuits, as this method is intuitive and computationally cheap.^31–35^ Yet, directly relating individual circuit elements to distinct physical processes is challenging — especially in PSCs due to their mixed ionic-electronic nature.^29^ As a result, the perovskite impedance field is shifting toward drift-diffusion (DD) models,^20,25,26,36^ where each input parameter has a distinct and unambiguous physical meaning. However, fitting to experimental impedance spectra is time-consuming and requires expertise in selecting the appropriate free parameters and their respective ranges.

To avoid this costly process, Bennett et al. propose an analytical model.^28^ While it offers many physical insights, it cannot yield impedance spectra with three or more features^26^ and is only valid for DC voltages near *V*_oc_.^28^

Alternatively, impedance spectra can be interpreted with standalone equations. This eliminates the need for doing any modeling. Such formulas have been proposed in the literature for PSC and directly relate the ion mobility to a characteristic frequency of the impedance spectrum and a thickness.^26,28,36,37^ However, the formulas yield different ion mobilities for the same impedance measurement, as the characteristic frequencies and thicknesses are not simply interchangeable.

Here, we derive a new (stand-alone) formula that directly links the LF feature in impedance spectroscopy to ionic motion. With extensive drift-diffusion simulations, we show that it can be used to obtain the ion mobility. The accuracy of other formulas from the literature is also assessed and found to be less effective. Lastly, we demonstrate how to apply the new formula to experimental data.

Figure 1 shows a typical impedance measurement of a MAPI PSC in the dark and under illumination. We find that illuminating the solar cell aids in lowering the overall impedance such that the ionic (LF) and electronic (HF) peaks are easily observed. In order to extract the ion mobility from the LF peak frequency (*f*_LF_), we proceed as follows. The response time τ of the ionic motion is related to *f*_LH_ by τ = 1/2π*f*_LF_. At the low frequency peak, the ions move over the total thickness *L*_tot_ of the PSC in response to the alternating field. So, *L*_tot_ = μ_ion_ *F*, where *F* is the electric field experienced by the ions. By approximating the electric field as *F* ≈ *V*_int_/*L*_tot_, we estimate the ion mobility as

1 $$\mu_{\mathrm{ion}}=2\pi f_{\mathrm{LF}}\frac{L_{\mathrm{tot}}^{2}}{V_{\mathrm{int}}}$$

where *V*_int_ is the internal voltage drop. *V*_int_ is approximated by *qV*_int_ ≈ Δ*E* – *qV*_DC_, where *V*_DC_ is the applied DC voltage in the IS measurement, and Δ*E* is the difference in the electrode work functions. Since it is reasonable to assume the electrodes form ohmic contacts with the transport layers (TLs), the conduction band of the ETL and/or the valence band of the HTL could be used instead.^28^ These values can be obtained from the literature and are determined via ultraviolet photoelectron spectroscopy, kelvin probe force microscopy, etc.^38^ If these values are not readily available, one could also approximate the internal voltage using the open-circuit voltage (*V*_oc_), *V*_int_ ≈ *V*_oc_ – *V*_DC_, as long as *V*_DC_ < *V*_oc_.

**Figure 1:**
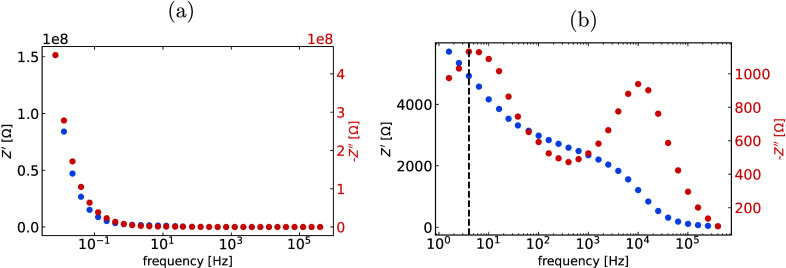
Impedance measurement of a MAPI PSC at 0 V and (a) in the dark and at (b) 1 sun equivalent. The dashed line indicates *f*_LF_. *Z*′ is the real part of impedance and *Z*″ its imaginary part.

When the DC voltage equals *V*_oc_, the above approximations for the internal voltage are no longer valid because the internal voltage across the TLs and perovskite approaches zero and diffusion dominates charge carrier transport rather than drift. In this scenario, the internal voltage can be approximated as $V_{\mathrm{int}}\approx \frac{\Delta E}{q}-0.4$ V. The offset between the energy levels and the internal voltage is to account for band bending as reported by Bartesaghi et al.^39,40^

We verify the formula for a wide variety of solar cells and multiple DC voltages, using the open-source drift-diffusion software SIMsalabim.^41^ For each physical quantity in Table S1, a value is randomly sampled within its specified range, resulting in a unique set of 38 parameters per simulated solar cell. We vary the layer thicknesses, band offsets, mobilities, trap densities, capture coefficients, permittivities, ion densities, etc. To the best of our knowledge, this is the first time such a large number of input variables has been sampled in drift-diffusion simulations. For example, in impedance drift-diffusion simulations it is usually assumed that the cations are mobile while the anions are immobile^20,26,36^ or anions are mobile and cations are immobile.^42^ Since both are possible, we randomly selected the mobile ion type.

With these input parameters, the solar cell’s steady-state current–voltage (*JV*) curve and impedance spectrum are generated. The steady-state *JV* curve is included only to demonstrate that a large number of different solar cells are covered with these input parameters (Figure S5). The PCEs of the simulated solar cells range from 1.1% to 28.7% with some even having an S-shaped *JV* curve.

The impedance spectrum provides *f*_LF_, which is used to compute the ion mobility via eq 1. The calculated ion mobility is then compared to the input ion mobility (and represents one point in Figure 2).

**Figure 2:**
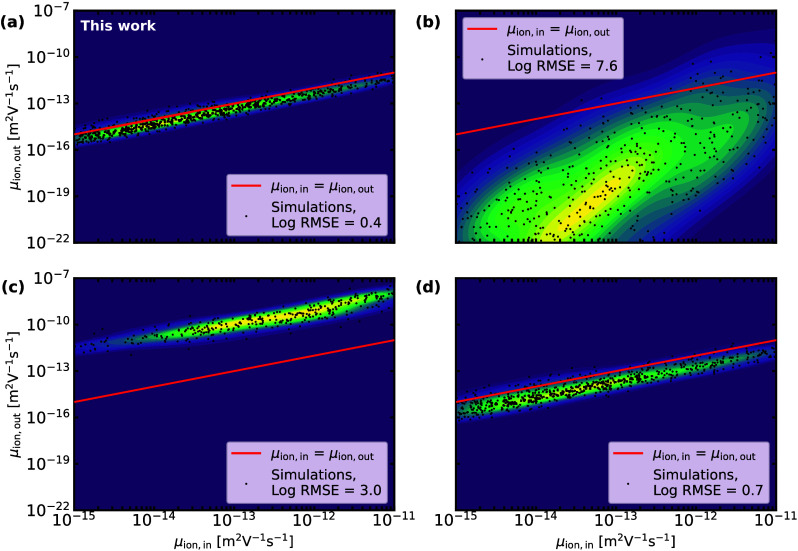
At 0 V and under 1 sun intensity the input ion mobility vs the ion mobility computed via the equations provided in (a) this work, (b) Sajedi Alvar et al., (c) Peng et al., and (d) Clarke et al.^26,36,37^ Each point represents one impedance spectrum. A kernel density estimate of the points is displayed in the background, computed using the scipy.stats.gaussian_kde python package.^44^ The same set of 594 impedance spectra served as input for (a), (b), and (d). For (b), not all points are visible, its minimum value is 1.1 × 10^–36^ m^2^ V^–1^ s^–1^. In (c), 320 impedance spectra are used, because the spectra are selected on *Z*_gap_^′′^ > 1%*Z*_LF_^′′^ instead of *Z*_gap_^′′^ ≤ 15%*Z*_LF_^′′^, and *f*_ankle_ can only be determined if the LF and HF features are overlapping.

However, not all generated impedance spectra are useful, e.g., some do not exhibit a LF and a HF peak. Also, they should fulfill the following conditions:

1. The LF peak should be at least 5% of the HF peak, and vice versa.
2. The LF peak should be positive ( $-Z_{\mathrm{LF}}^{\prime \prime }$ > 0).
3. The height of the local minimum between the LF and HF peak should be smaller than or equal to 15% of the LF peak.

The conditions delineate for which impedance spectra in eq 1 works. With the first condition, we exclude cases where there are very few ions in the PSCs as it would also be difficult experimentally to identify the ionic peak if it is very small. When the LF peak dominates the impedance spectrum, screening of the internal electric field is very strong which is outside the scope of our claim.The other two conditions ensure the characteristic frequency of purely the ionic relaxation process is measured. If the LF and HF peak overlap, the relaxation process of the LF peak is dispersive, meaning the observed characteristic frequency deviates from the true ionic characteristic frequency, as it is a combination of ionic and electronic relaxation processes.

All included impedance spectra are shown in Figure S6. More information about the included impedance spectra and the numerical method in general can be found in the Supporting Information.

Figure 2a shows the relationship between the input and output ion mobility of our formula for 594 impedance spectra. It clearly demonstrates that our formula captures the underlying physics, because the output ion mobility matches the input ion mobility very closely. When *V*_DC_ = 0 V, the ion mobility is estimated within a factor of 0.4 below and 2.5 above the true ion mobility. The factor is determined via the logarithmic root-mean-square error (RMSE). (The logarithmic RMSE is employed instead of the standard RMSE, as μ_ion,in_ spans several orders of magnitude. Without the logarithm, errors at 1 × 10^–15^ would be overshadowed by those at 1 × 10^–11^ m^2^ V^–1^ s^–1^, leading to an imbalanced assessment of the error. The factor is computed as 10^–RMSE^ and 10^+RMSE^.) The spread can be reduced by not allowing ions to migrate into the ETL and/or HTL. We randomly select whether the ions can migrate into these layers, though this process is usually excluded from drift-diffusion simulations as it is not well understood.^43^ We still observe the LF peak in most cases, yet *f*_LF_ tends to shift slightly when ions enter one or both TLs, leading to more spread in Figure 2.

Also, for the 0.5 and 1.0 V DC voltages, the spread is smaller, and both estimate the ion mobility within a factor 0.5 below and 2 above the true ion mobility (see Figures S7a and S8a). For *V*_oc_, the ion mobility is estimated to be a factor of 0.3 below and 3 above the true ion mobility (see Figure S9a). Thus, our formula accurately determines the ion mobility within half an order of magnitude across all DC voltages ranging from 0 V to *V*_oc_, demonstrating its effectiveness and offering an improvement over the current state-of-the-art.

As mentioned before, several other formulas have been proposed in the literature to determine the ion mobility from impedance spectroscopy.^26,28,36,37^ Sajedi Alvar et al. relate the ion mobility to the device’s total thickness and *f*_gap_,^36^ after rewriting:

2 $$\mu_{\mathrm{ion}}=2\pi \cdot V_{T}^{-1}\cdot \frac{f_{\mathrm{gap}}^{5}}{f_{\mathrm{HF}}^{4}}\cdot L_{\mathrm{tot}}^{2}$$

where $V_{T}=\frac{k_{B}T}{q}$ is the thermal voltage.

Peng et al. identify an ionic chemical diffusion coefficient, *D*_μ_, and relate it to the perovskite thickness (*L*_perov_) and the frequency at the “ankle” of the LF and HF arc (ω_d_) in the Nyquist plot:^37^

$$\omega_{d}=D_{\mu }\cdot L_{\mathrm{perov}}^{-2}$$

and after rewriting

3 $$\mu_{\mathrm{ion}}=2\pi \cdot V_{T}^{-1}f_{\mathrm{ankle}}L_{\mathrm{perov}}^{2}$$

*f*_ankle_ is equivalent to *f*_gap_ if and only if the LF and HF features are overlapping (see Figure S3b).

Clarke et al. use an analytical model to interpret experimental impedance spectra of PSCs.^26^ In the model the appearance of the LF feature (*f*_LF_) is linked to the Debye length and is, when rearranged, given by

4 $$\begin{array}{ll} \mu_{\mathrm{ion}} & =\lambda_{D}\cdot f_{\mathrm{LF}}\cdot L_{\mathrm{perov}}\\ & =\sqrt{\frac{\varepsilon_{\mathrm{perov}}}{qV_{T}N_{\mathrm{mobile}\mathrm{ion}}}}\cdot f_{\mathrm{LF}}\cdot L_{\mathrm{perov}} \end{array}$$

where λ_D_ is the Debye length, ε_perov_ is the permittivity of the perovskite, and *N*_mobile ion_ is the mobile ion density.

To compare our formula with these formulas, we reuse the generated impedance spectra and parameter sets and extract, e.g., *f*_gap_, *f*_HF_, and *L*_tot_, when using eq 2, instead of *f*_LF_, *V*_int_ and *L*_tot_. (The formula from Bennett et al. is not included in the analysis because it requires multiple parameters that are difficult to measure experimentally, e.g., the ion-vacancy density and d*Q*_DC_/d*V*_DC_ — the derivative of the ionic space-charge density with respect to the applied DC voltage.^28^)

Figure 2 also presents the relationship between the input and the output ion mobility of these formulas and indicates that there exists a discrepancy for eqs 2 and 3.

For eq 2 this is understandable, as a PSC does not fulfill the formula’s underlying assumptions. The equation is obtained from a model developed by Bandara and Mellander, which is intended for ionic liquids and electrolytes, and in the model it is assumed that the sample is cylindrical.^34^

For eq 3 the ion mobility is determined over 3 orders of magnitude, albeit consistently overestimated. Adjusting the factor 2π to 1/120 leads to a substantial reduction in the standard deviation (Log RMSE = 0.5). However, it cannot be lower than the standard deviation of our formula because *f*_ankle_ does not reflect a single relaxation process, but a frequency where the ionic and electronic relaxation processes combine.

The use of *f*_LF_ is based on how the mobile ions respond to the applied sinusoidal voltage wave: at frequencies before the LF feature, they can follow the wave; at frequencies above the LF feature, they remain fixed. At the frequencies in between, the mobile ions partially respond but lag behind, thereby screening the internal electric field, causing phase-delayed recombination,^26^ and affecting the extraction of electronic charge carriers. This causes the current wave to lag behind the voltage wave, which is reflected in the imaginary part of the impedance — as it represents the phase shift between the voltage and current wave. Therefore, *f*_LF_ is related to the ion mobility, as it reflects where the mobile ions barely keep up with the voltage wave. Schmidt et al. observe experimentally that *f*_LF_ is directly proportional to ion conductivity, which is in turn directly related to the ion mobility.^20^ Riquelme et al. also find with the DD-software IonMonger that *D*_ion_ is directly proportional to *f*_LF_,^30^ and not to *f*_ankle_, *f*_gap_ or *f*_HF_.

In eq 4 the ion mobility is determined with *f*_LF_, giving significantly higher accuracy (see Figures 2, S7, S8, and S9). Although this formula approximates the input ion diffusion coefficient with half an order of magnitude, its practical application is limited, since it requires the mobile ion concentration, which is challenging to measure experimentally.

Apart from the LF and HF features, PSCs may also exhibit a midfrequency (MF) feature in their impedance response.^26,29^ So far, in the drift-diffusion simulations, we considered only one mobile ion species. This results in impedance spectra featuring only LF and HF features (with sometimes a small inductive MF feature).

If an additional mobile ion species is added to the perovskite in the drift-diffusion simulations, at sufficiently high concentration, their movement appears as a distinct feature in the impedance spectrum (Figure S4). In other words, we observe both a LF *and* a MF feature when both the anion and cation density are significantly high and their mobilities are significantly different. In this scenario, both ion mobilities can be extracted from the impedance spectrum by using *f*_LF_ directly and by replacing *f*_LF_ with the characteristic frequency of the MF peak (*f*_MF_) in eq 1. Therefore, we suggest that some *but not all* observed MF features in the impedance spectra of PSCs are caused by an additional mobile ion species or vacancy in the perovskite.

Now, we demonstrate how to apply eq 1 to experimental impedance spectra. For this purpose, we prepared a PSC with the structure ITO/MeO-2PACz/MAPI/C_60_/SnO_2_/Cu, where ITO stands for indium tin oxide, MeO-2PACz for (2-(3,6-dimethoxy-9*H*-carbazol-9-yl)­ethyl)­phosphonic acid and MAPI for methylammonium lead iodide. The thickness of this device is *L*_tot_ = 882 nm. The impedance spectrum is measured under illumination such that the impedance is no longer dominated by the geometrical capacitance. The critical requirement is to avoid dark conditions. Further details on the device materials, device preparation, and impedance measurement procedure are provided in the Supporting Information.

Our formula, just like the other formulas, cannot be applied to all impedance spectra. As shown in Figure 1b, at *V*_DC_ = 0 V, condition 3 is not fulfilled. Since the observed imaginary impedance spectrum is the sum of the LF and HF peaks, if the LF peak merges with the HF peak, the observed characteristic frequency deviates from its true value. The conditions ensure that the ionic relaxation process occurs independently, so the observed characteristic frequency matches the true value, allowing the ion mobility to be calculated with adequate accuracy.

Nevertheless, eq 1 may still be applied to this spectrum provided one accepts a significantly higher uncertainty in the estimated ion mobility. A more robust strategy is to measure impedance at multiple DC voltages, as the LF and HF peak often decouple at a certain voltage.

Here, the spectrum measured at *V*_DC_ = 0.8 V fulfills the conditions and gives a characteristic frequency of *f*_LF_ = 4 Hz (see Figure 3). To compute the internal voltage, the work function of the anode and lowest unoccupied molecular orbital (LUMO) of the ETL are used in this case and are *E*_f,ITO_ = −4.7 eV,^45^ and *E*_LUMO,C60_ = −3.85 eV,^25^ respectively. *E*_LUMO,C60_ can be used instead of the Fermi level of Cu, because — as mentioned before — it is reasonable to assume the electrodes form ohmic contacts with the TLs.^28^ Using the above-mentioned values in eq 1, we obtain an ion mobility of 4 × 10^–10^ m^2^ V^–1^ s^–1^ (*D*_ion_ = 1 × 10^–11^ m^2^ s^–1^).

**Figure 3:**
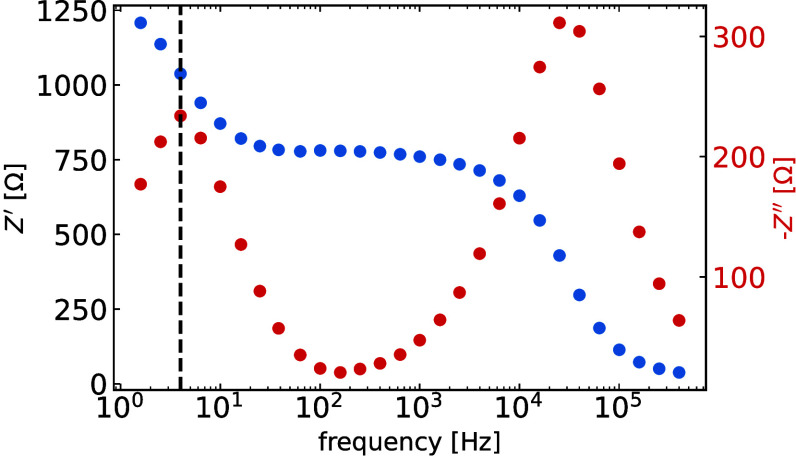
Impedance measurement at 0.8 V and 1 sun eq for a MAPI PSC. *Z*′ is the real part of impedance and *Z*″ is its imaginary part. The black dashed line indicates the characteristic frequency of the low-frequency peak and is 4 Hz in this case.

To conclude, a new formula is introduced to determine the ion mobility of a complete perovskite solar cell from its impedance response (eq 1). The formula uses the characteristic frequency of the low-frequency feature, the solar cell’s internal voltage and the solar cell’s thickness. We show using drift-diffusion software that the formula determines the ion mobility within half an order of magnitude and is applicable even to poorly performing solar cells. For this purpose, we generate more than 500 impedance spectra for multiple DC voltages, where the solar cells differ in trap densities, ion densities, layer thicknesses, band offsets, mobilities, capture coefficients, permittivities, etc.

Alternative formulas from the literature to determine the ion mobility are also validated but show limited accuracy or require the mobile ion concentration, which is challenging to measure experimentally.

Furthermore, the appearance of a low- *and* midfrequency feature may be explained with the new formula: two types of *mobile* defects coexist at the same time, a possibility that is underexamined in the literature.

Lastly, an experimental impedance measurement is conducted to deduce the ion mobility of a MAPI PSC, yielding a value of 4 × 10^–10^ m^2^ V^–1^ s^–1^. The advantage of determining the ion mobility via impedance spectroscopy is that it is precise, and with the formula, the interpretation is straightforward.

## Supplementary Material



## Notes

Drift-diffusion simulation parameters and method; Experimental methods for device materials, device preparation, and impedance measurements; Simulated *JV* curves and impedance spectra; Histograms of sampled parameter space; Simulated input vs output ion mobility for multiple DC voltages and alternative formulas (PDF)
