# Supplementary material for: Determining the Ion Mobility in Perovskite Solar Cells from Impedance Spectroscopy
Source: ACS Energy Lett. 2025 Aug 26;10(9):4574–9. doi: 10.1021/acsenergylett.5c01690 (PMC12442514; doi:10.1021/acsenergylett.5c01690)
Supplement: Supplementary file 1 [file nz5c01690_si_001.pdf]

# Supporting Information

## Determining the Ion Mobility in Perovskite Solar Cells from Impedance Spectroscopy

Fransien D. Elhorst,[†] Javier E. Sebastián Alonso,[‡] Henk J. Bolink,[‡] L. Jan Anton Koster[*,†]

† *Zernike Institute for Advanced Materials, University of Groningen, Nijenborgh 3, 9747 AG Groningen, The Netherlands*

‡ *Instituto de Ciencia Molecular, Universidad de Valencia, C/ Catedrático J. Beltrán 2, 46980 Paterna, Spain*

* Corresponding author. E-mail: l.j.a.koster@rug.nl

# Numerical method

The simulated impedance spectra are generated with the open-source drift-diffusion software SIMsalabim.[1] Like other drift-diffusion models, SIMsalabim is based on the three fundamental differential equations governing charge transport in a semiconductor: the transport equations, the continuity equations and the Poisson equation. For PSCs, these fundamental equations are extended to incorporate not only electrons and holes but also anions and cations. The extension is essential to model hysteresis in JV curves,[2] and the LF peak in impedance spectra.[3] Further details of the drift-diffusion software are provided by Koopmans *et al.*,[1] and Heester *et al.*[4]

In all impedance simulations, the device is exposed to the AM1.5G spectrum at 1 sun illumination intensity. Under illumination, the device's resistance against changes in current flow decreases, making the LF peak detectable.
As mentioned in the main text, to validate formula 1, it is necessary to demonstrate that the formula holds over a wide variety of generated impedance spectra. In other words, the characteristic frequency $f_{\mathrm{LF}}$ (see Figure S3) should scale with the dependent variables — $\mu_{\mathrm{ion}}$, $L_{\mathrm{tot}}$, $\Delta E$ and $V_{\mathrm{DC}}$ — while remaining independent of other parameters. The independent variables are: ion concentration, bulk trap density, interface trap density, band offset, electron and hole mobility, etc. By fixing $E_{\mathrm{c,perov}} = 3.9$ eV and $E_{\mathrm{v,perov}} = 5.5$ eV, the band gap — a key electronic characteristic of a (MAPI) perovskite solar cell — is preserved in the DD simulations. All parameters that are randomly sampled are indicated in Table S1. Consequently, it is not naturally expected that the calculated ion mobility ($\mu_{\mathrm{ion,out}}$) will match the input ion mobility ($\mu_{\mathrm{ion,in}}$).

We apply a standard algorithm to compare $\mu_{\mathrm{ion,in}}$ to $\mu_{\mathrm{ion,out}}$ for each generated impedance spectrum. In the algorithm we:

1. Randomly sample a value between the lower and upper limit listed in Table S1 for all 38 device parameters
2. Store the input ion mobility $\mu_{\text{ion,in}}$, solar cell thickness $L_{\text{tot}}$, and the difference in fermi-level (work function) of the electrodes $\Delta E$
3. Compute the internal voltage using $qV_{\text{int}} \approx \Delta E - qV_{\text{DC}}$ for $V_{\text{DC}} = 0$ V, 0.5 V and 1.0 V; and $V_{\text{int}} \approx \frac{\Delta E}{q} - 0.4\text{V}$ for $V_{\text{oc}}$
4. Predict $\mu_{\text{ion,in}}$ from $f_{\text{LF}}$ using equation 1 and set the frequency interval from $f_{\text{min}} = f_{\text{LF,pred}} \cdot 10^{-2}$ Hz to $f_{\text{max}} = 10^6$ Hz
5. Use SIMsalabim to generate the impedance spectra corresponding with the 38 randomly sampled device parameters
6. Smoothen the impedance spectrum with the Gaussian filter from the `scipy.ndimage` python package,[5] where sigma is set to 1
7. Find the characteristic frequency of the LF peak of the smoothened impedance spectra using the `scipy.signal.argrelextrema` python package[5]
8. Compute the output ion mobility as
   $\mu_{\text{ion,out}} = 2\pi V_{\text{int}}^{-1} f_{\text{LF}} L_{\text{tot}}^2$

This procedure is repeated to retrieve $\mu_{\text{ion,in}}$ and $\mu_{\text{ion,out}}$ for hundreds of impedance spectra. However, not all simulations generate realistic and useful impedance spectra. As the device parameters are randomly sampled over a broad interval, some of the simulations do not converge within the allocated time of 5 minutes. Others have a lot of noise, large error bars, less than two impedance peaks, etc.

Only impedance spectra that exhibit two impedance peaks in the imaginary domain can be included in the analysis. Subsequently, we set the numerical requirement that spectra that exhibit large error bars, are only included if the minimum and maximum frequency, where the LF peak could possibly occur, are at maximum a factor 30 apart (see Figure S1).

Afterwards, we apply the conditions that are described in the main text. These conditions apply for experimental and simulated impedance spectra alike:

1. $0.05 < \frac{Z''_{\text{LF}}}{Z''_{\text{HF}}} < \frac{1}{0.05} = 20$

2. $-Z''_{\text{LF}} > 0$

3. $-Z''_{\text{gap}} \leq -0.15 Z''_{\text{LF}}$

We continue generating new impedance spectra until there are more than 500 that fulfill the conditions for each DC voltage. All included impedance spectra are shown in Figure S6, and their corresponding JV curves in Figure S5. Figures S10, S11, S12 and S13 show that all input parameters exhibit more or less a uniform distribution across the whole parameter range — with the exception of the anion & cation density. Thus the parameter space as mentioned in Table S1, is uniformly explored for all but one parameter.

That the ion density histogram is non-uniform, is not surprising. An impedance spectrum can only give an ion mobility when both a LF and a HF peak appear. If the ion density is too low, the LF is so small that it is not visible. If the ion density is too high, the HF is so small compared to the LF peak that it is not visible.

Figure S2 shows when setting as the only requirement that the spectrum should have two peaks, the ion density histogram is still not uniform. Thus, not the selection criteria lead to a narrowing of the ion density range, but this is an inherent property of the method itself.

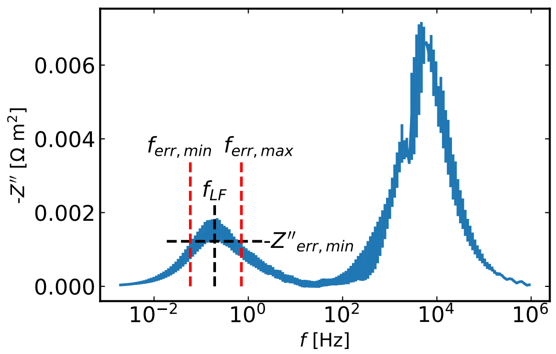


Figure S1: Some of the simulated impedance spectra show large error bars around the LF peak. Therefore the LF peak might not actually occur at $f_{\mathrm{LF}}$, but anywhere between $f_{err,min}$ and $f_{err,max}$. To find these bounds, first the lower limit of $-Z''_{LF}$, $-Z''_{err,min}$, is drawn. Than $f_{err,min}$ and $f_{err,max}$ are located, this is where the upper limit of $-Z''$ drops just below $-Z''_{err,min}$ on either side of $f_{LF}$. For the spectrum to be included in the analysis, we require $f_{err,max} \leq 10^{1.5} \cdot f_{err,min} \approx 30 \cdot f_{err,min}$.

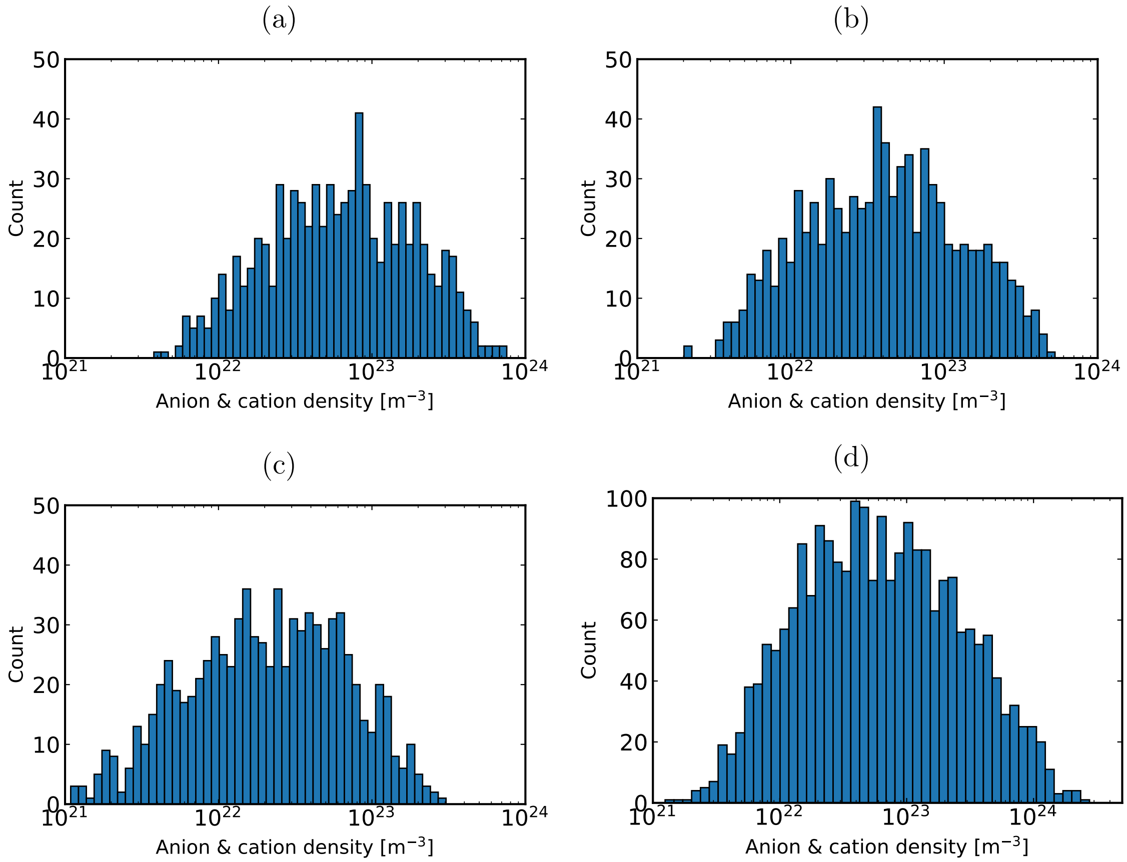


Figure S2: Histogram of the anion & cation density including all impedance spectra that show a LF and HF peak, when $V_{\mathrm{DC}}$ is a) 0 V, b) 0.5 V, c) 1.0 V, d) $V_{\mathrm{oc}}$.

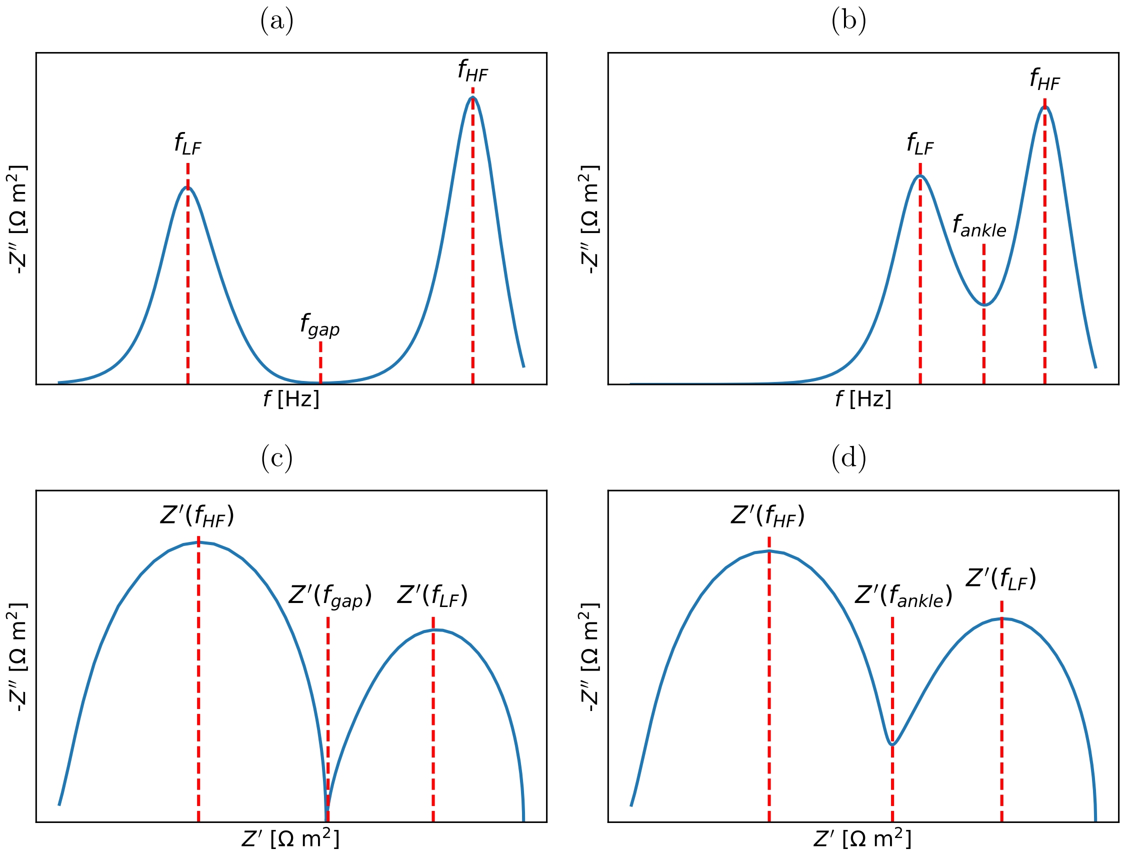


Figure S3: a) Sketch of the imaginary part of impedance as function of frequency and c) the corresponding Nyquist plot, as typically observed for PSCs. b) Sketch of the imaginary part of impedance as function of frequency and d) the corresponding Nyquist plot, when the low-frequency (LF) and high-frequency (HF) peak overlap. The characteristic frequencies are marked. $f_{\mathrm{LF}}$, $f_{\mathrm{gap}}$, and $f_{\mathrm{HF}}$ correspond to the low-frequency peak, the local minimum in $-Z''$ between the LF and HF peak, and the high-frequency peak, respectively. Note the distinction between $f_{\mathrm{gap}}$ and $f_{\mathrm{ankle}}$, where $f_{\mathrm{gap}} = f_{\mathrm{ankle}}$ but $f_{\mathrm{ankle}} \neq f_{\mathrm{gap}}$ as $f_{\mathrm{ankle}}$ is defined if and only if the LF and HF features overlap.

## Device materials

All materials were used as received without further purification. Photolithographically patterned ITO-coated glass substrates were purchased from Naranjo Substrates. Methylammonium iodide (MAI, >99.0%), (2-(3,6-Dimethoxy-9H-carbazol-9-yl)ethyl)phosphonic acid (MeO-2PACz, >99.95%), were purchased from Luminescence Technology Corp. Fullerene $C_{60}$ (>99.95%) was purchased from Creaphys GmbH. Lead(II) iodide ($PbI_2$, 99.999%, trace metals basis) in form of beads was purchased from Alfa Aesar.

## Device preparation

ITO-coated glass substrates were subsequently cleaned with soap, water and isopropanol in an ultrasonic bath, followed by 15 min UV-ozone treatment. The substrates were transferred to a vacuum chamber integrated in a nitrogen-filled glovebox and evacuated to a pressure of $\times 10^{-6}$ mbar for the charge extraction layers' deposition. The deposition rate for the MeO-2PACz was 0.2 Å/s. The methylammonium leadiodide perovskite films (850 nm) were deposited on these substrates by co-evaporation of the methylammonion iodide and lead iodide precursors in a third, dedicated vacuum chamber. Both sublimation sources have individual QCM sensors and an additional one is positioned close to the substrates for the overall deposition rate measurement. All sources were individually calibrated for their respective materials and no cross-reading between different materials is ensured by the position of the sources, shutters and sensors. During the $MAPbI_3$ perovskite deposition, the individual QCM deposition rates were: 2.0 Å/s for $PbI_2$ and for 2.4 Å/s MAI and the pressure of the chamber was below $5 \times 10^{-6}$ mbar and the substrates were kept at room temperature.[6] Following the perovskite layer a thin layer (10 nm) of $C_{60}$ was deposited using a sublimation rate of 0.5 Å/s. The $SnO_2$ layer (20 nm) was deposited via atomic layer deposition (ALD) at room temperature using an Arradiance's GEMStar XT Thermal ALD system integrated into

an $N_2$-filled glovebox, following the procedure already published by us elsewhere.[7] For $SnO_2$ deposition, the ALD chamber is heated to 60 ºC, the bottle containing the Sn precursor tetrakis(dimethylamino)tin (TDAT) was heated to 60 ºC, and the bottle of water (oxidizer) was not heated. The precursor manifolds were heated to 115 and 140 ºC, respectively. The $SnO_2$ deposition process consists of a series of purges of TDAT for 550 ms and water vapor for 200 ms, each of them followed by $N_2$ purges to clear off the precursors from the ALD chamber. The metal copper (Cu) electrode (100 nm) was deposited in a second vacuum chamber using aluminum boats as sources, by applying currents ranging from 2.0 to 3.0 A.

## Impedance measurements

All measurements were conducted at room temperature under ambient air, using a home-built sample holder within a Gamry Calibration Shield. Before each impedance measurement the sample was first pre-biased for 30 seconds at the DC voltage and illumination intensity of interest. Impedance measurements were performed with a Gamry Interface 1000 potentiostat, applying a voltage amplitude of 30 mV. A LUXEON CoB Core Range white LED was employed to illuminate the solar cells. The light intensity was set to 1 sun eq. using a calibrated silicon reference cell.

# Results

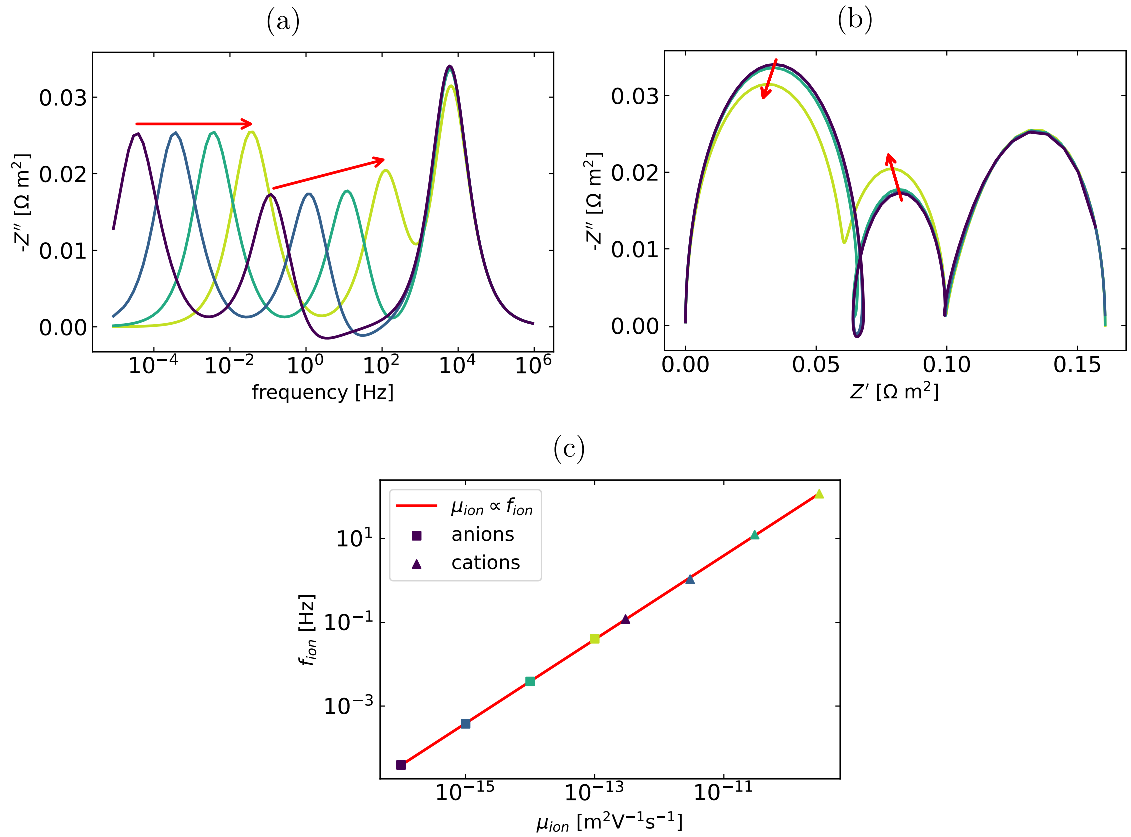


Figure S4: a) Simulated imaginary parts of the impedance spectra for increasing anion and cation mobilities where $\mu_{\text{anion}} \neq \mu_{\text{cation}}$ (see Table S2 for the input parameters). b) Corresponding Nyquist plot. c) The corresponding anion and cation mobilities versus the characteristic frequency of the low and mid frequency peaks, respectively. In this case, using $f_{\text{LF}}$ in eq 1 gives the anion mobility, replacing $f_{\text{LF}}$ by $f_{\text{MF}}$ in eq 1 gives the cation mobility.

Table S1: Parameter ranges in the drift-diffusion simulations

| **Parameter** | **Symbol** | **Lower limit** | **Upper limit** | **Unit** |
|---|---|---|---|---|
| *Layer parameters* | | | | |
| Illumination side[a] | | ETL | HTL | |
| Perovskite thickness | $L_{\text{perov}}$ | $200 \times 10^{-9}$ | $800 \times 10^{-9}$ | m |
| ETL thickness | $L_{\text{ETL}}$ | $20 \times 10^{-9}$ | $50 \times 10^{-9}$ | m |
| HTL thickness | $L_{\text{HTL}}$ | $20 \times 10^{-9}$ | $50 \times 10^{-9}$ | m |
| Perovskite relative permittivity | $\varepsilon_{\text{perov}}$ | 20 | 60 | |
| ETL relative permittivity | $\varepsilon_{\text{ETL}}$ | 3 | 15 | |
| HTL relative permittivity | $\varepsilon_{\text{HTL}}$ | 3 | 15 | |
| Perovskite effective density of states | $N_{\text{C,perov}}$ | $1 \times 10^{24}$ | $1 \times 10^{25}$ | $\text{m}^{-3}$ |
| ETL effective density of states | $N_{\text{C,ETL}}$ | $1 \times 10^{25}$ | $5 \times 10^{26}$ | $\text{m}^{-3}$ |
| HTL effective density of states | $N_{\text{C,HTL}}$ | $1 \times 10^{25}$ | $5 \times 10^{26}$ | $\text{m}^{-3}$ |
| ETL donor doping density | $N_{\text{D,ETL}}$ | $1 \times 10^{20}$ | $5 \times 10^{24}$ | $\text{m}^{-3}$ |
| HTL acceptor doping density | $N_{\text{A,HTL}}$ | $1 \times 10^{20}$ | $5 \times 10^{24}$ | $\text{m}^{-3}$ |
| *Ions* | | | | |
| Ion type[a] | | Mobile anions & immobile cations | Mobile cations & immobile anions | |
| Anion & cation density[b] | $N_{\text{ion}}$ | $1 \times 10^{20}$ | $1 \times 10^{24}$ | $\text{m}^{-3}$ |
| Mobile ion mobility | $\mu_{\text{ion}}$ | $1 \times 10^{-15}$ | $1 \times 10^{-11}$ | $\text{m}^2\ \text{V}^{-1}\ \text{s}^{-1}$ |
| Ions may enter ETL | | Yes | No | |
| Ions may enter HTL | | Yes | No | |

*Trapping & transport of electron and holes*

| | | | | |
|---|---|---|---|---|
| Bulk trap type[a] | | Acceptor | Donor | |
| Bulk trap density | $N_{\mathrm{t,bulk}}$ | $1 \times 10^{18}$ | $1 \times 10^{22}$ | $\mathrm{m^{-3}}$ |
| Bulk electron capture coefficient | $C_{\mathrm{n,bulk}}$ | $1 \times 10^{-15}$ | $1 \times 10^{-13}$ | $\mathrm{m^3\ s^{-1}}$ |
| Bulk hole capture coefficient | $C_{\mathrm{p,bulk}}$ | $1 \times 10^{-15}$ | $1 \times 10^{-13}$ | $\mathrm{m^3\ s^{-1}}$ |
| Energy level of bulk traps | $E_{\mathrm{t,bulk}}$ | 4.01 | 5.39 | eV |
| | | | | |
| ETL/perov interface trap type[a] | | Acceptor | Donor | |
| HTL/perov interface trap type[a] | | Acceptor | Donor | |
| ETL/perov interface trap density | $N_{\mathrm{t,int,ETL}}$ | $1 \times 10^{11}$ | $1 \times 10^{13}$ | $\mathrm{m^{-2}}$ |
| HTL/perov interface trap density | $N_{\mathrm{t,int,HTL}}$ | $1 \times 10^{11}$ | $1 \times 10^{13}$ | $\mathrm{m^{-2}}$ |
| ETL/perov interface electron capture coefficient | $C_{\mathrm{n,int,ETL}}$ | $1 \times 10^{-15}$ | $1 \times 10^{-13}$ | $\mathrm{m^3\ s^{-1}}$ |
| ETL/perov interface hole capture coefficient | $C_{\mathrm{p,int,ETL}}$ | $1 \times 10^{-15}$ | $1 \times 10^{-13}$ | $\mathrm{m^3\ s^{-1}}$ |
| HTL/perov interface electron capture coefficient | $C_{\mathrm{n,int,HTL}}$ | $1 \times 10^{-15}$ | $1 \times 10^{-13}$ | $\mathrm{m^3\ s^{-1}}$ |
| HTL/perov interface hole capture coefficient | $C_{\mathrm{p,int,HTL}}$ | $1 \times 10^{-15}$ | $1 \times 10^{-13}$ | $\mathrm{m^3\ s^{-1}}$ |
| Energy level of ETL/perov interface traps | $E_{\mathrm{t,int,ETL}}$ | 4.01 | 5.39 | eV |
| Energy level of HTL/perov interface traps | $E_{\mathrm{t,int,HTL}}$ | 4.01 | 5.39 | eV |
| | | | | |
| Perovskite electron mobility | $\mu_{\mathrm{n,perov}}$ | $1 \times 10^{-5}$ | $1 \times 10^{-3}$ | $\mathrm{m^2\ V^{-1}\ s^{-1}}$ |

| | | | | |
|---|---|---|---|---|
| Perovskite hole mobility | $\mu_{\mathrm{p,perov}}$ | $1 \times 10^{-5}$ | $1 \times 10^{-3}$ | $\mathrm{m^2\ V^{-1}\ s^{-1}}$ |
| ETL electron mobility | $\mu_{\mathrm{n,ETL}}$ | $1 \times 10^{-7}$ | $1 \times 10^{-5}$ | $\mathrm{m^2\ V^{-1}\ s^{-1}}$ |
| HTL hole mobility | $\mu_{\mathrm{p,HTL}}$ | $1 \times 10^{-7}$ | $1 \times 10^{-5}$ | $\mathrm{m^2\ V^{-1}\ s^{-1}}$ |
| *Band offset* | | | | |
| ETL conduction band energy level and its electrode's work function | $E_{\mathrm{CB,ETL}} = E_{\mathrm{f}}$ | 3.8 | 4 | eV |
| HTL valence band energy level and its electrode's work function | $E_{\mathrm{VB,HTL}} = E_{\mathrm{f}}$ | 5.4 | 5.6 | eV |

[a] For these parameters, the lower and upper "limits" represent discrete choices rather than a range, where one of the two values is randomly selected.

[b] When $V_{\mathrm{oc}}$ is the operating point, the anion & cation density range is instead set to $1 \times 10^{21}$ - $5 \times 10^{24}$ $\mathrm{m^{-3}}$. Since at $V_{\mathrm{oc}}$ higher ion concentrations are necessary to observe the low-frequency peak.

Table S2: Device parameters used in the drift-diffusion simulation of Figure S4.

| **Parameter** | **Symbol** | **ETL** | **Perovskite** | **HTL** | **Unit** |
|---|---|---|---|---|---|
| Thickness | $L$ | $25 \times 10^{-9}$ | $400 \times 10^{-9}$ | $10 \times 10^{-9}$ | m |
| Relative permittivity | $\varepsilon_r$ | 5 | 24 | 3 | |
| Effective density of states | $N_C$ | $5 \times 10^{26}$ | $2.2 \times 10^{24}$ | $1 \times 10^{26}$ | $m^{-3}$ |
| Donor (acceptor) doping density | $N_{D(A)}$ | 0 | 0 | 0 | $m^{-3}$ |
| Ions allowed in this layer | | No | Yes | No | |
| Anion concentration | $N_a$ | 0 | $1 \times 10^{22}$ | 0 | $m^{-3}$ |
| Cation concentration | $N_c$ | 0 | $5 \times 10^{22}$ | 0 | $m^{-3}$ |
| Bulk trap density | $N_{t,bulk}$ | 0 | $1 \times 10^{20}$ | 0 | $m^{-3}$ |
| Trap density with interface to the right | $N_{t,int}$ | $4 \times 10^{12}$ | $1 \times 10^{12}$ | - | $m^{-3}$ |
| Energy level of traps in the bulk and at the interfaces | $E_t$ | 4.7 | 4.7 | 4.7 | eV |
| Electron (hole) capture coefficients in the bulk and at the interfaces | $C_{n(p)}$ | $2 \times 10^{-14}$ | $2 \times 10^{-14}$ | $2 \times 10^{-14}$ | $m^3\ s^{-1}$ |
| Band-to-band recombination constant | $k$ | $1 \times 10^{-17}$ | $1 \times 10^{-17}$ | $1 \times 10^{-17}$ | $m^3\ s^{-1}$ |
| Electron mobility | $\mu_n$ | $1 \times 10^{-6}$ | $1 \times 10^{-4}$ | $1 \times 10^{-9}$ | $m^2\ V^{-1}\ s^{-1}$ |
| Hole mobility | $\mu_p$ | $1 \times 10^{-9}$ | $1 \times 10^{-4}$ | $1 \times 10^{-7}$ | $m^2\ V^{-1}\ s^{-1}$ |
| Conduction band energy level | $E_{CB}$ | 3.9 | 3.9 | 2.9 | eV |
| Valence band energy level | $E_{VB}$ | 6.5 | 5.5 | 5.5 | eV |

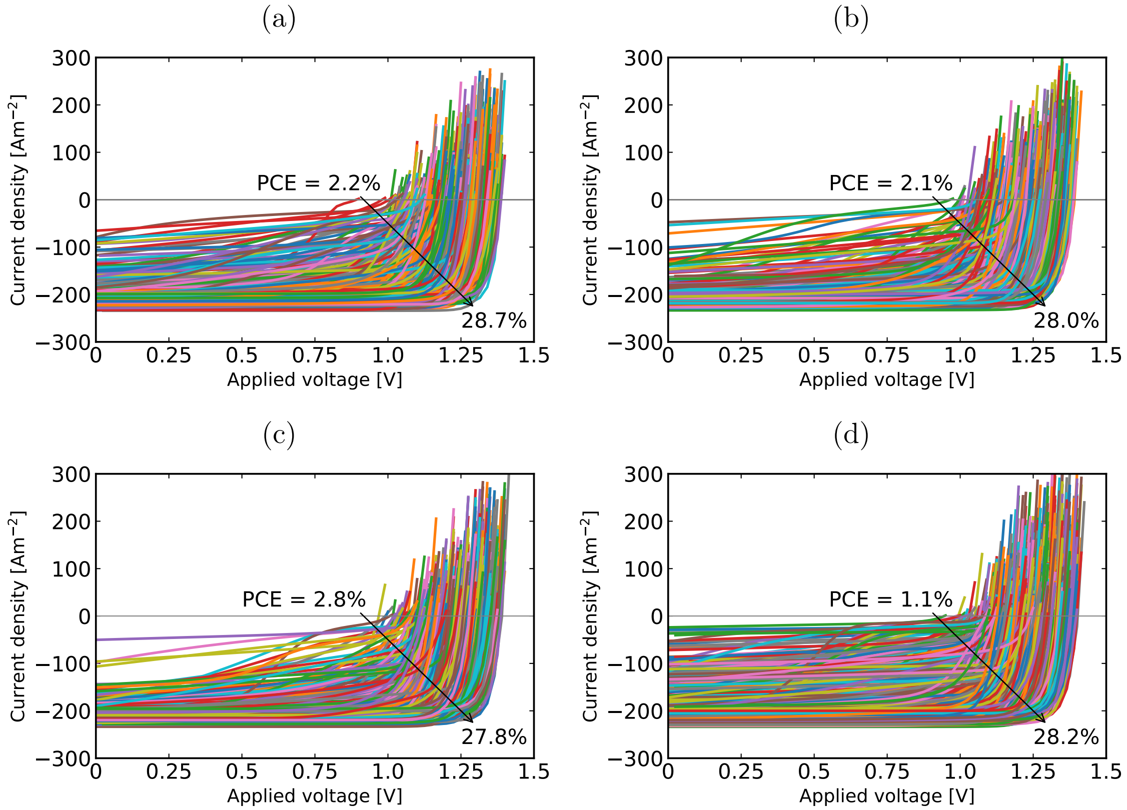


Figure S5: Generated steady-state JV curves using the same unique sets of numbers as the impedance spectra in Figure S6. Since new unique sets are created for each DC voltage in the impedance simulations, the JV curves differ per DC voltage. The JV curves correspond to the unique sets generated for the impedance spectra with DC voltage operating points: a) 0 V, b) 0.5 V, c) 1.0 V, and d) $V_{\mathrm{oc}}$.

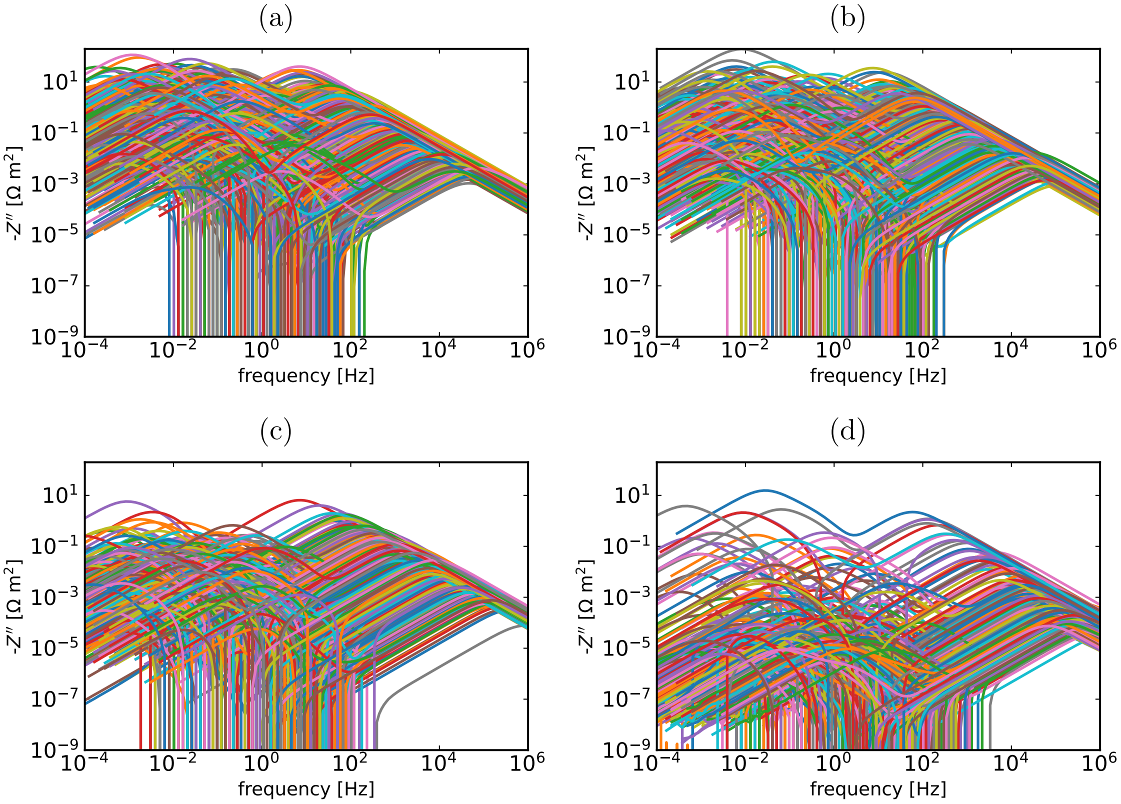


Figure S6: Generated impedance spectra for DC voltage operating points: a) 0 V, b) 0.5 V, c) 1.0 V, and d) $V_{\mathrm{oc}}$. Several impedance spectra exhibit an inductive region between the LF and HF feature, causing the vertical drop in the imaginary part of impedance — an artifact of log-log plotting, which omits negative values.

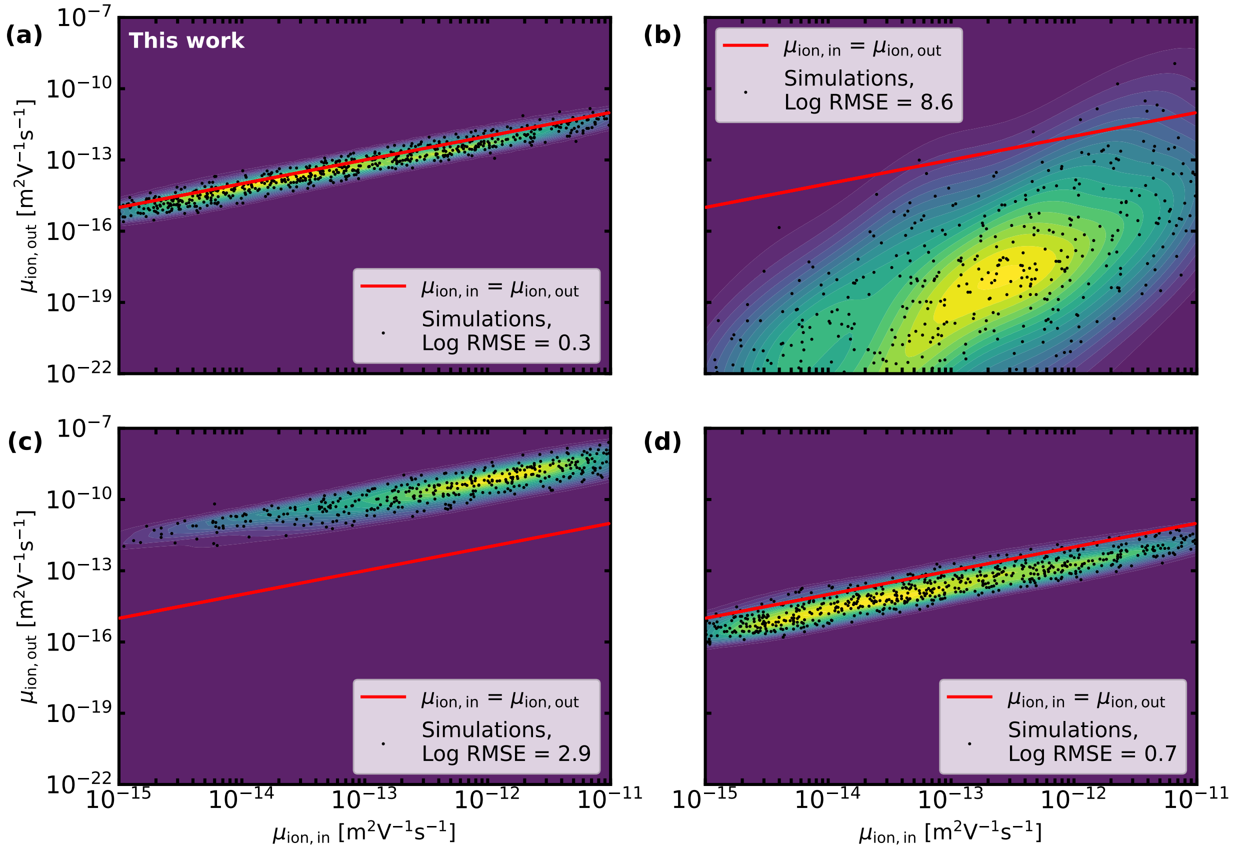


Figure S7: At 0.5 V and under 1 sun of illumination the input ion mobility versus the ion mobility computed via the equations provided in a) this work, b) Sajedi Alvar *et al.* c) Peng *et al.* d) Clarke *et al.*[8–10] Each point represents one impedance spectra. A kernel density estimate of the points is displayed in the background, computed using the `scipy.stats.gaussian_kde` python package.[5] The same set of 602 impedance spectra served as input for (a), (b), and (d). For (b) not all points are visible, its minimum value is $6.7 \times 10^{-35}$ m$^2$V$^{-1}$s$^{-1}$. In (c) 369 impedance spectra are used, because the spectra are selected on $Z''_{\text{gap}} > 1\%\ Z''_{\text{LF}}$ instead of $Z''_{\text{gap}} \leq 15\%\ Z''_{\text{LF}}$.

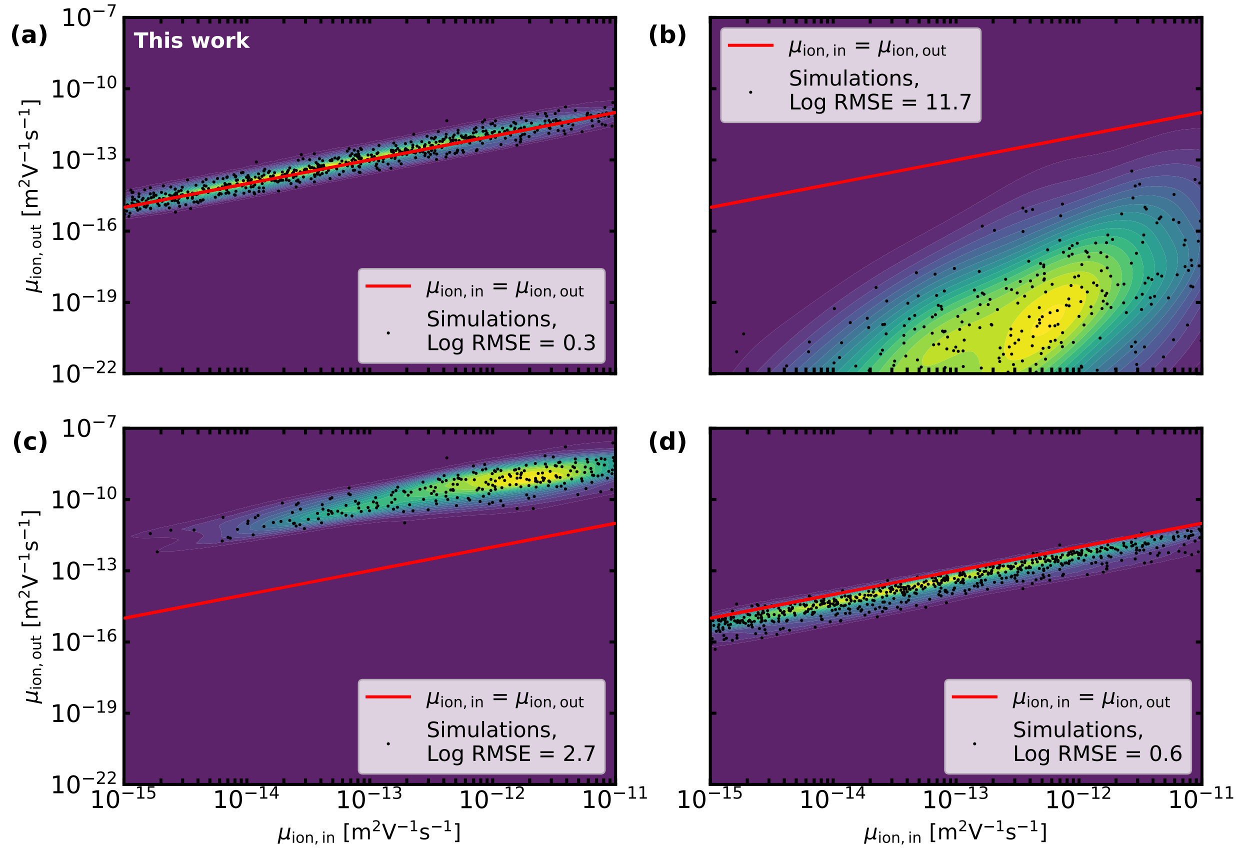


Figure S8: At 1.0 V and under 1 sun of illumination the input ion mobility versus the ion mobility computed via the equations provided in a) this work, b) Sajedi Alvar *et al.* c) Peng *et al.* d) Clarke *et al.*[8–10] Each point represents one impedance spectra. A kernel density estimate of the points is displayed in the background, computed using the `scipy.stats.gaussian_kde` python package.[5] The same set of 588 impedance spectra served as input for (a), (b), and (d). For (b) not all points are visible, its minimum value is $1.9 \times 10^{-40}$ $m^2V^{-1}s^{-1}$. In (c) 244 impedance spectra are used, because the spectra are selected on $Z''_{\mathrm{gap}} > 1\%\ Z''_{\mathrm{LF}}$ instead of $Z''_{\mathrm{gap}} \leq 15\%\ Z''_{\mathrm{LF}}$.

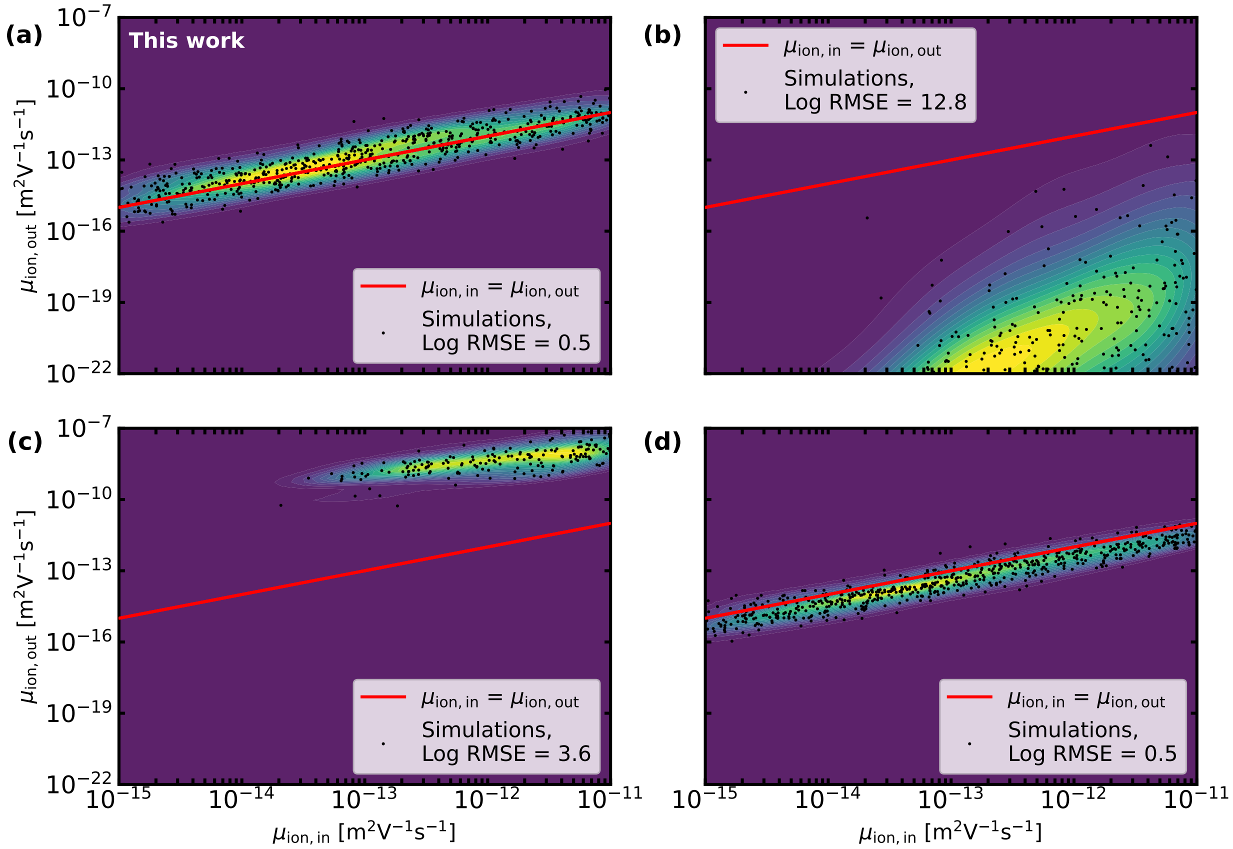


Figure S9: At $V_{oc}$ and under 1 sun of illumination the input ion mobility versus the ion mobility computed via the equations provided in a) this work, b) Sajedi Alvar *et al.* c) Peng *et al.* d) Clarke *et al.*[8–10] Each point represents one impedance spectra. A kernel density estimate of the points is displayed in the background, computed using the `scipy.stats.gaussian_kde` python package.[5] The same set of 597 impedance spectra served as input for (a), (b), and (d). For (b) not all points are visible, its minimum value is $3.1 \times 10^{-40}$ $m^2V^{-1}s^{-1}$. In (c) 176 impedance spectra are used, because the spectra are selected on $Z''_{gap} > 1\%\ Z''_{LF}$ instead of $Z''_{gap} \leq 15\%\ Z''_{LF}$.

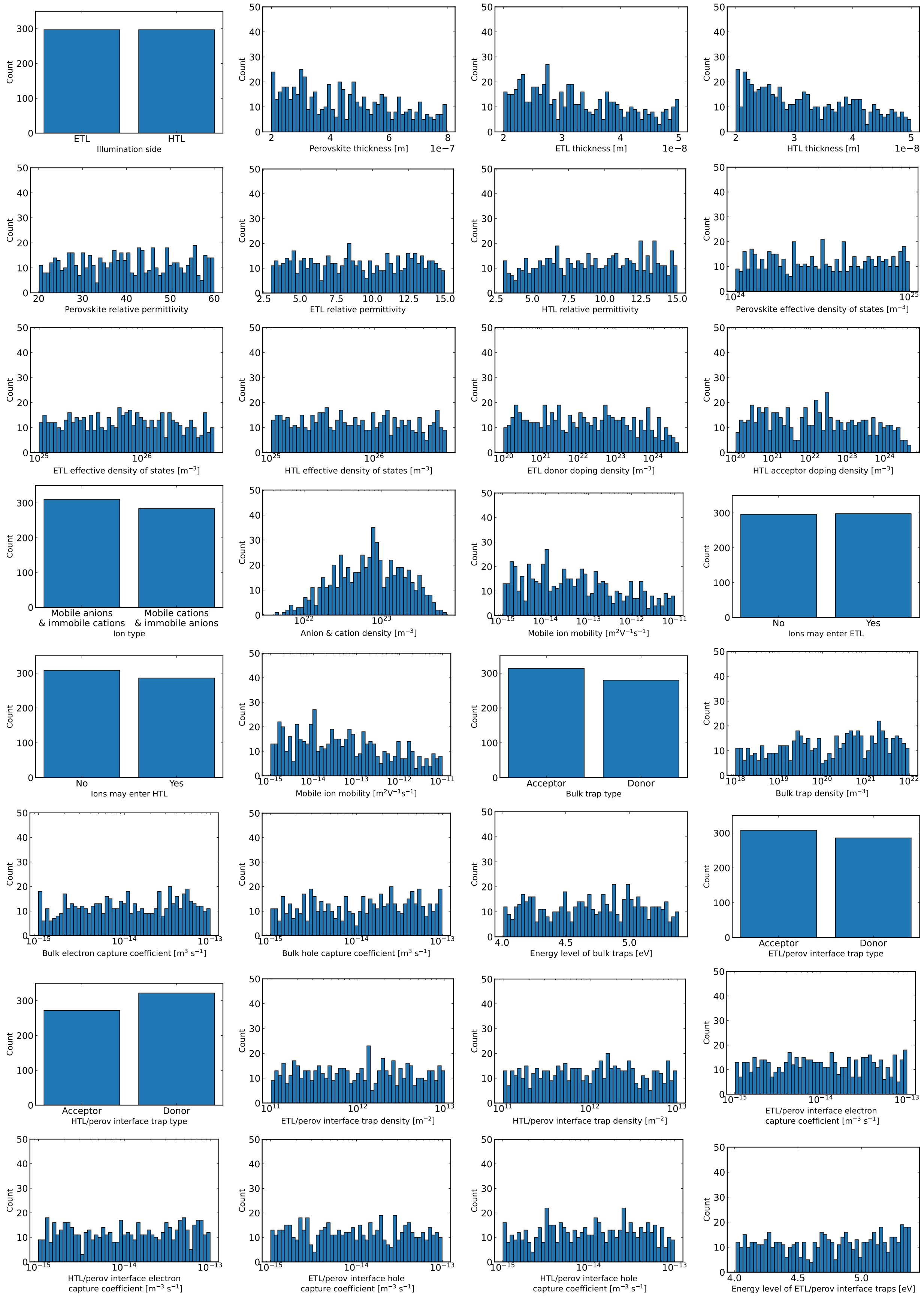
Count
ETL
HTL
Illumination side
Perovskite thickness [m]
1e−7
ETL thickness [m]
1e−8
HTL thickness [m]
1e−8
Perovskite relative permittivity
ETL relative permittivity
HTL relative permittivity
Perovskite effective density of states [m$^{-3}$]
ETL effective density of states [m$^{-3}$]
HTL effective density of states [m$^{-3}$]
ETL donor doping density [m$^{-3}$]
HTL acceptor doping density [m$^{-3}$]
Mobile anions & immobile cations
Mobile cations & immobile anions
Ion type
Anion & cation density [m$^{-3}$]
Mobile ion mobility [m$^2$V$^{-1}$s$^{-1}$]
No
Yes
Ions may enter ETL
No
Yes
Ions may enter HTL
Mobile ion mobility [m$^2$V$^{-1}$s$^{-1}$]
Acceptor
Donor
Bulk trap type
Bulk trap density [m$^{-3}$]
Bulk electron capture coefficient [m$^3$ s$^{-1}$]
Bulk hole capture coefficient [m$^3$ s$^{-1}$]
Energy level of bulk traps [eV]
Acceptor
Donor
ETL/perov interface trap type
Acceptor
Donor
HTL/perov interface trap type
ETL/perov interface trap density [m$^{-2}$]
HTL/perov interface trap density [m$^{-2}$]
ETL/perov interface electron capture coefficient [m$^{-3}$ s$^{-1}$]
HTL/perov interface electron capture coefficient [m$^{-3}$ s$^{-1}$]
ETL/perov interface hole capture coefficient [m$^{-3}$ s$^{-1}$]
HTL/perov interface hole capture coefficient [m$^{-3}$ s$^{-1}$]
Energy level of ETL/perov interface traps [eV]

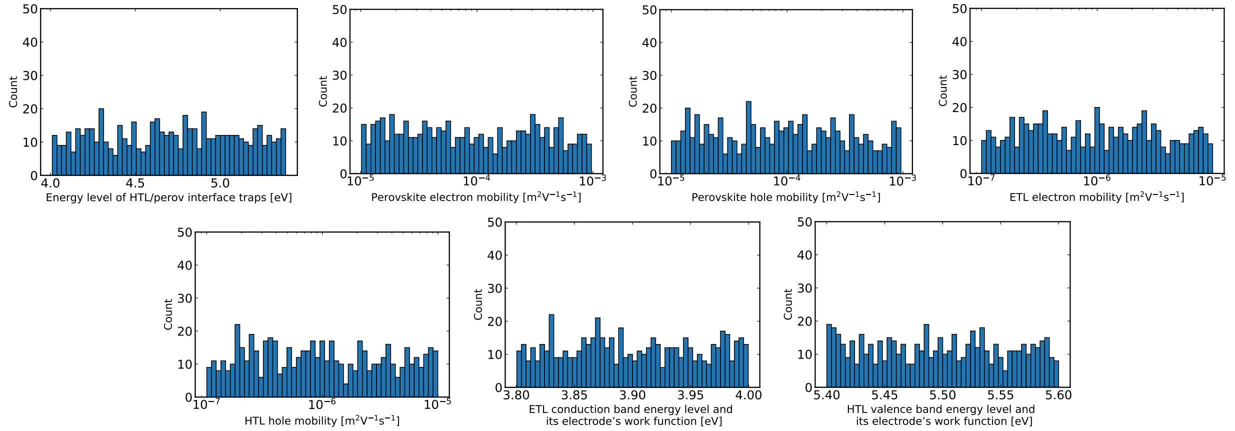


Figure S10: The sampled parameter space for $V_{\mathrm{DC}} = 0$ V. Each subfigure shows per parameter indicated in Table S1, the amount of times a (range of) value(s) occurs.

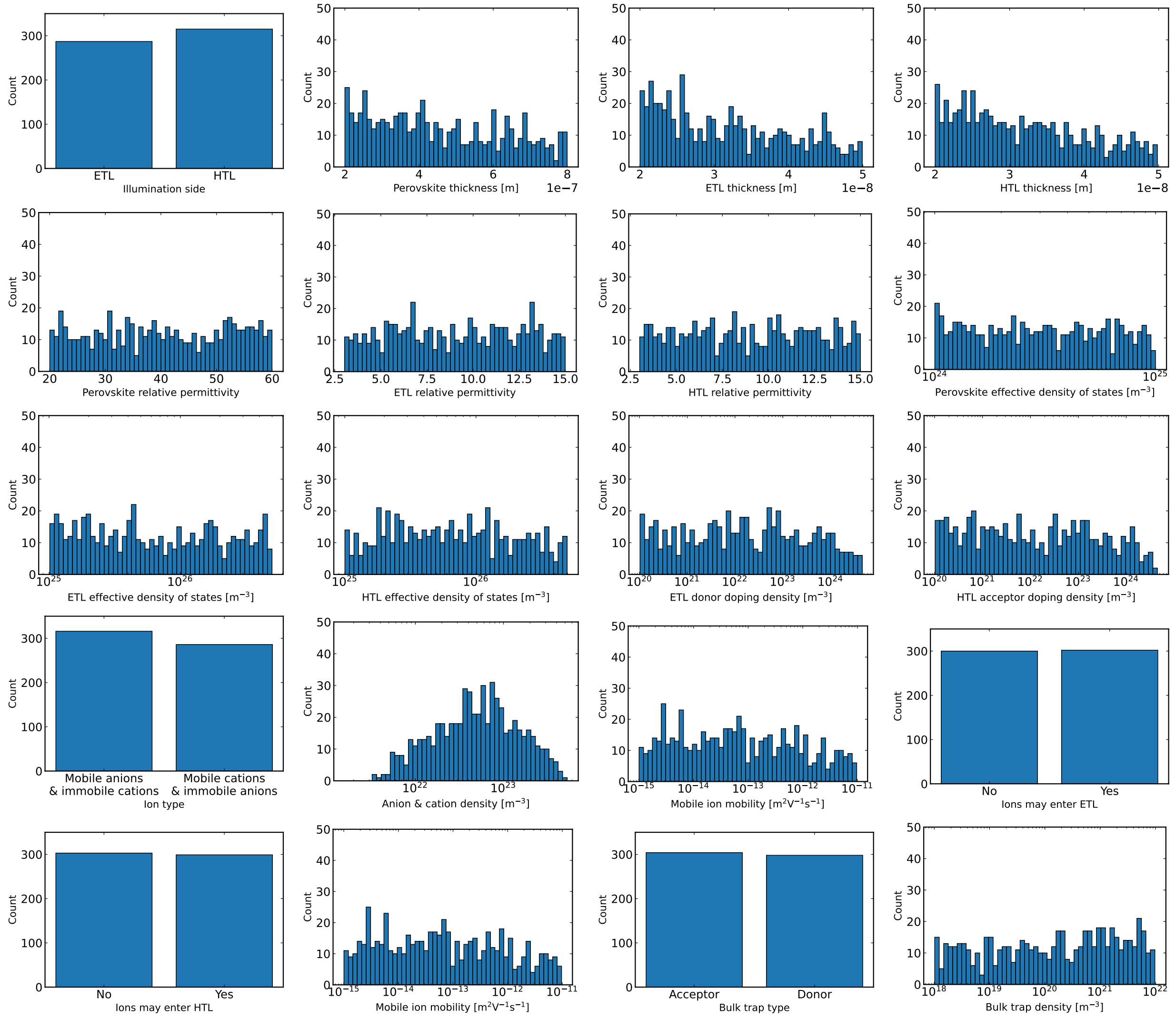

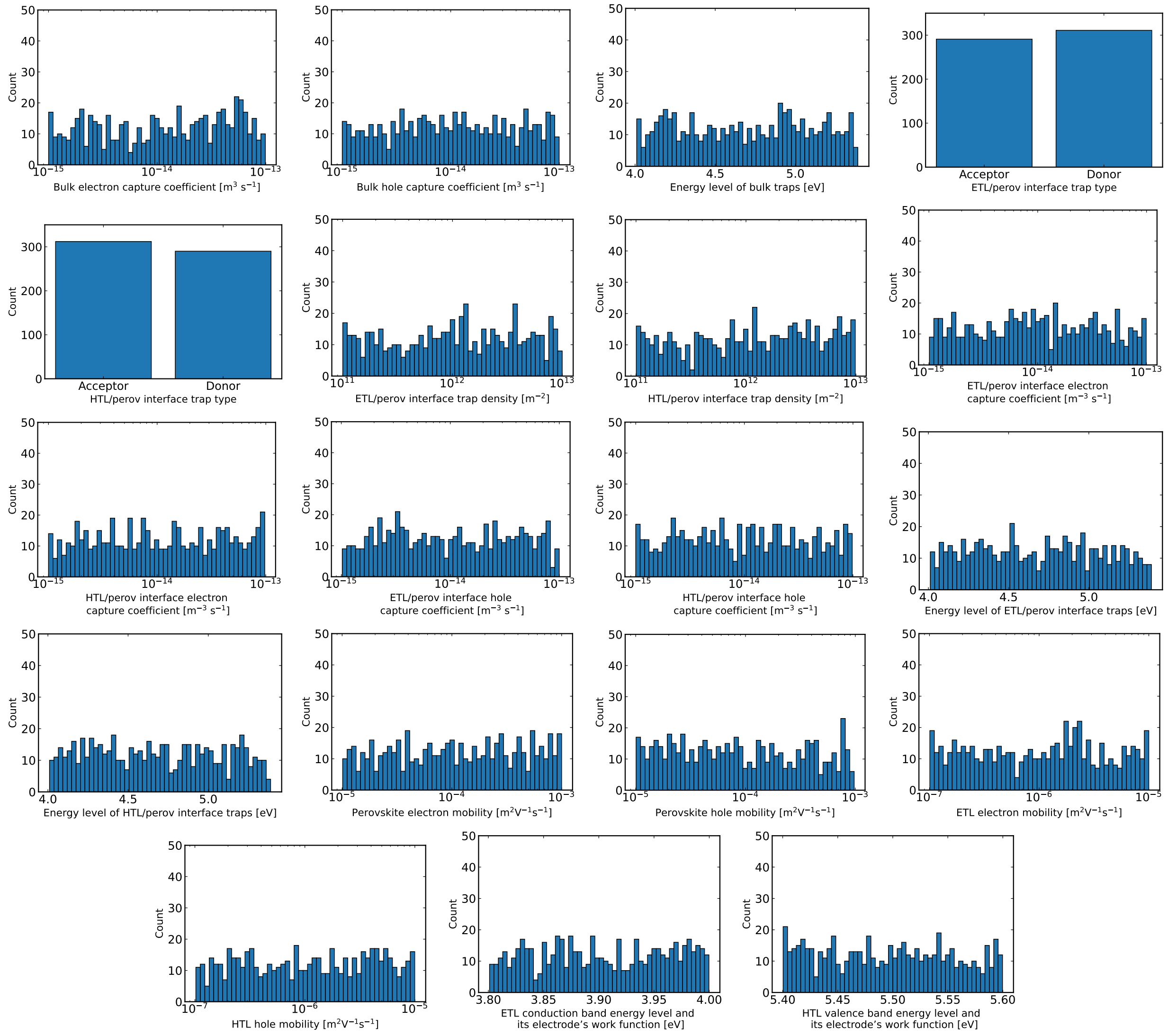


Figure S11: The sampled parameter space for $V_{\mathrm{DC}} = 0.5$ V. Each subfigure shows per parameter indicated in Table S1, the amount of times a (range of) value(s) occurs.

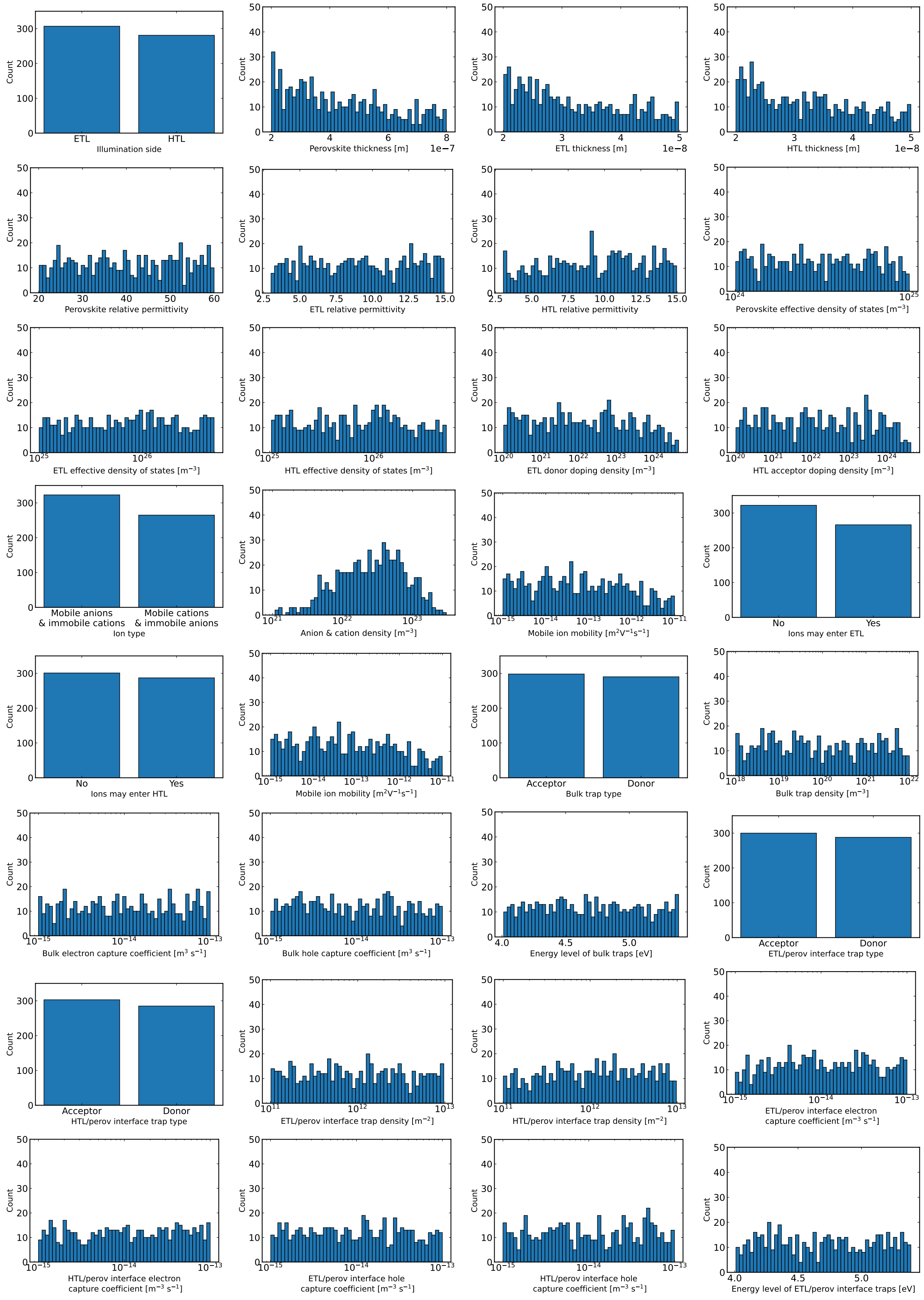
Count
ETL
HTL
Illumination side
Perovskite thickness [m]
1e−7
ETL thickness [m]
1e−8
HTL thickness [m]
1e−8
Perovskite relative permittivity
ETL relative permittivity
HTL relative permittivity
Perovskite effective density of states [m$^{-3}$]
ETL effective density of states [m$^{-3}$]
HTL effective density of states [m$^{-3}$]
ETL donor doping density [m$^{-3}$]
HTL acceptor doping density [m$^{-3}$]
Mobile anions & immobile cations
Mobile cations & immobile anions
Ion type
Anion & cation density [m$^{-3}$]
Mobile ion mobility [m$^2$V$^{-1}$s$^{-1}$]
No
Yes
Ions may enter ETL
No
Yes
Ions may enter HTL
Mobile ion mobility [m$^2$V$^{-1}$s$^{-1}$]
Acceptor
Donor
Bulk trap type
Bulk trap density [m$^{-3}$]
Bulk electron capture coefficient [m$^3$ s$^{-1}$]
Bulk hole capture coefficient [m$^3$ s$^{-1}$]
Energy level of bulk traps [eV]
Acceptor
Donor
ETL/perov interface trap type
Acceptor
Donor
HTL/perov interface trap type
ETL/perov interface trap density [m$^{-2}$]
HTL/perov interface trap density [m$^{-2}$]
ETL/perov interface electron capture coefficient [m$^{-3}$ s$^{-1}$]
HTL/perov interface electron capture coefficient [m$^{-3}$ s$^{-1}$]
ETL/perov interface hole capture coefficient [m$^{-3}$ s$^{-1}$]
HTL/perov interface hole capture coefficient [m$^{-3}$ s$^{-1}$]
Energy level of ETL/perov interface traps [eV]

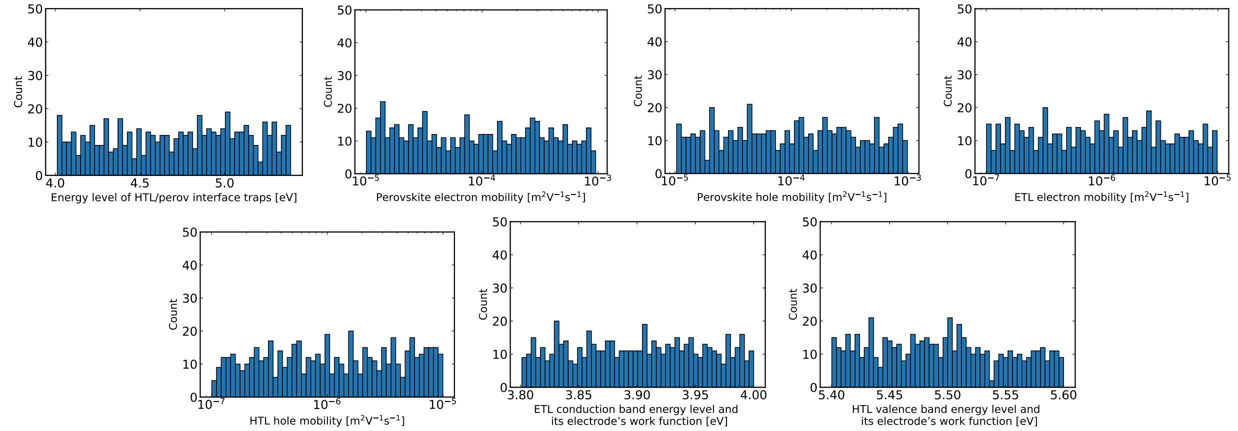


Figure S12: The sampled parameter space for $V_{\mathrm{DC}} = 1.0$ V. Each subfigure shows per parameter indicated in Table S1, the amount of times a (range of) value(s) occurs.

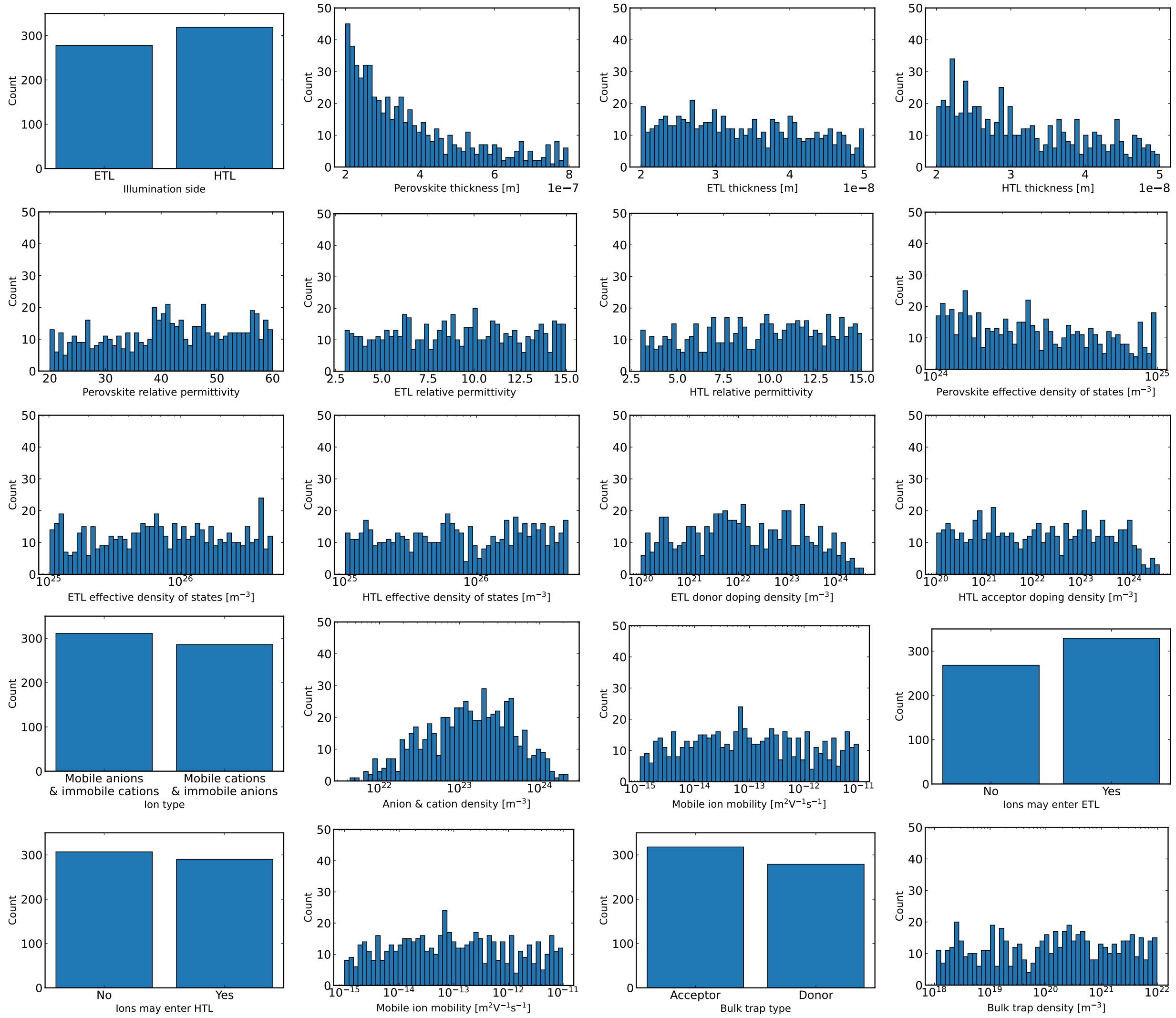

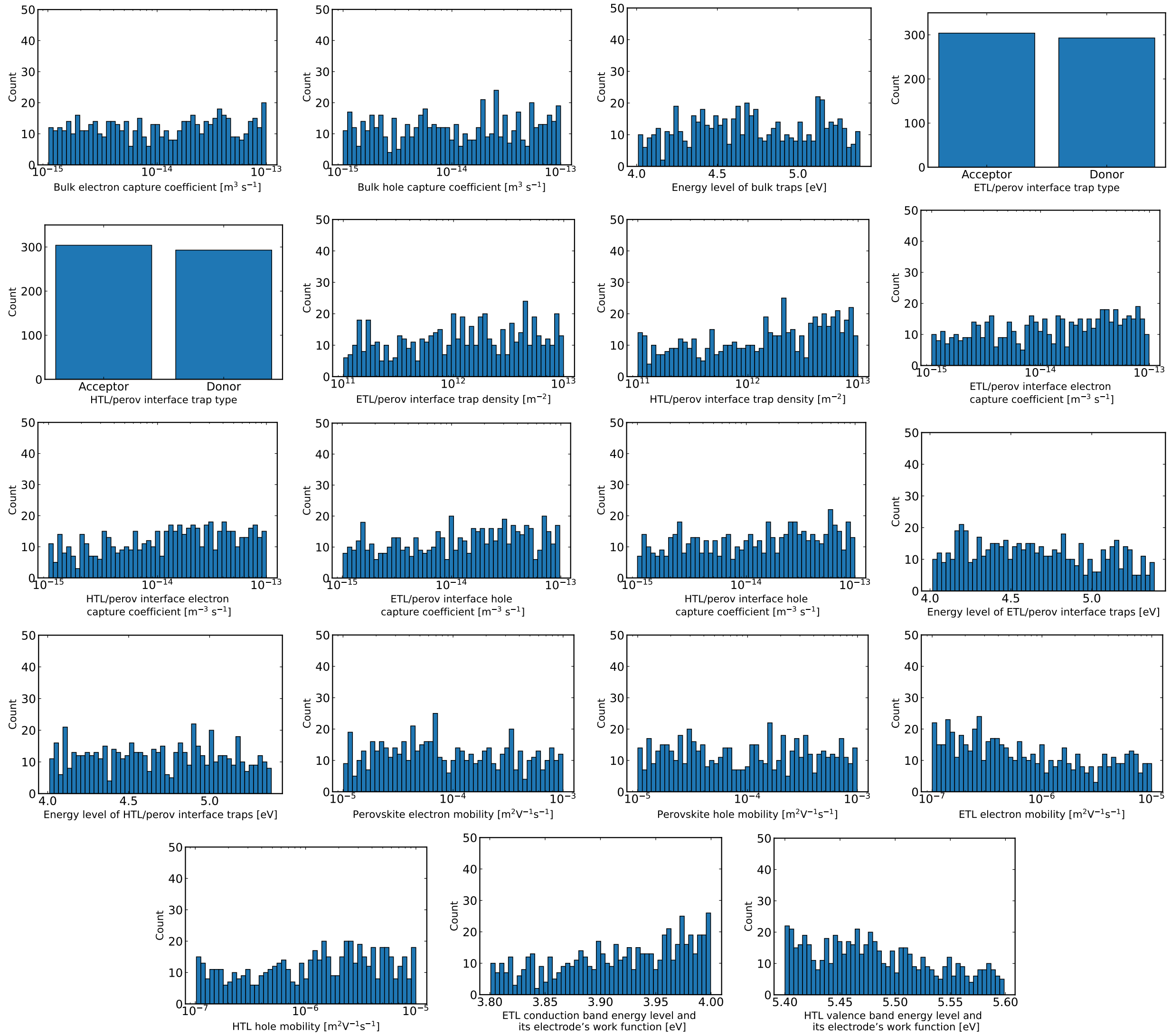


Figure S13: The sampled parameter space for $V_{\mathrm{DC}} = \mathrm{V_{oc}}$. Each subfigure shows per parameter indicated in Table S1, the amount of times a (range of) value(s) occurs.

# References


(1) Koopmans, M.; Le Corre, V. M.; Koster, L. J. A. SIMsalabim: An open-source drift-diffusion simulator for semiconductor devices. *Journal of Open Source Software* **2022**, *7*, 3727.

(2) Neukom, M. T.; Schiller, A.; Züfle, S.; Knapp, E.; Ávila, J.; Pérez-del-Rey, D.; Dreessen, C.; Zanoni, K. P. S.; Sessolo, M.; Bolink, H. J.; Ruhstaller, B. Consistent device simulation model describing perovskite solar cells in steady-state, transient, and frequency domain. *ACS Applied Materials & Interfaces* **2019**, *11*, 23320–23328.

(3) Riquelme, A.; Bennett, L. J.; Courtier, N. E.; Wolf, M. J.; Contreras-Bernal, L.; Walker, A. B.; Richardson, G.; Anta, J. A. Identification of recombination losses and charge collection efficiency in a perovskite solar cell by comparing impedance response to a drift-diffusion model. *Nanoscale* **2020**, *12*, 17385–17398.

(4) Heester, S.; Elhorst, F.; Martin Fernandez, P.; Le Corre, V.; Koopmans, M.; Koster, L. pySIMsalabim: a Python package to extend drift-diffusion modelling with SIMsalabim (submitted).

(5) Virtanen, P. et al. SciPy 1.0: fundamental algorithms for scientific computing in Python. *Nature Methods* **2020**, *17*, 261–272.

(6) Zanoni, K. P. S.; Martínez-Goyeneche, L.; Dreessen, C.; Sessolo, M.; Bolink, H. J. Photovoltaic devices using sublimed methylammonium lead iodide perovskites: long-term reproducible processing. *Solar RRL* **2023**, *7*, 2201073.

(7) Zanoni, K. P. S.; Pérez-del Rey, D.; Dreessen, C.; Rodkey, N.; Sessolo, M.; Soltanpoor, W.; Morales-Masis, M.; Bolink, H. J. Tin(IV) oxide electron transport layer via industrial-scale pulsed laser deposition for planar perovskite solar cells. *ACS Applied Materials & Interfaces* **2023**, *15*, 32621–32628.

(8) Sajedi Alvar, M.; Blom, P. W. M.; Wetzelaer, G.-J. A. Device model for methylammonium lead iodide perovskite with experimentally validated ion dynamics. *Advanced Electronic Materials* **2020**, *6*, 1900935.

(9) Peng, W.; Aranda, C.; Bakr, O. M.; Garcia-Belmonte, G.; Bisquert, J.; Guerrero, A. Quantification of ionic diffusion in lead halide perovskite single crystals. *ACS Energy Letters* **2018**, *3*, 1477–1481.

(10) Clarke, W.; Richardson, G.; Cameron, P. Understanding the full zoo of perovskite solar cell impedance spectra with the standard drift-diffusion model. *Advanced Energy Materials* **2024**, *14*, 2400955.
